# Identification of a basement membrane-based risk scoring system for prognosis prediction and individualized therapy in clear cell renal cell carcinoma

**DOI:** 10.3389/fgene.2023.1038924

**Published:** 2023-02-03

**Authors:** Yanlin Tang, Chujin Ye, Jiayi Zeng, Ping Zhu, Shouyu Cheng, Weinan Zeng, Bowen Yang, Yanjun Liu, Yuming Yu

**Affiliations:** ^1^ Shantou University Medical College, Shantou, China; ^2^ Department of Urology, Guangdong Provincial People's Hospital (Guangdong Academy of Medical Sciences), Southern Medical University, Guangzhou, China; ^3^ The Second School of Clinical Medicine, Southern Medical University, Guangzhou, China; ^4^ Department of Immunology, School of Basic Medical Science, Southern Medical University, Guangzhou, China; ^5^ School of Medicine, South China University of Technology, Guangzhou, China; ^6^ Guangdong Cardiovascular Institute, Guangdong Provincial People's Hospital, Guangdong Academy of Medical Sciences, Guangzhou, China

**Keywords:** clear cell renal cell carcinoma, basement membrane, gene expression, tumor microenvironment, individualized therapy

## Abstract

Clear cell renal cell carcinoma (ccRCC) belongs to one of the 10 most frequently diagnosed cancers worldwide and has a poor prognosis at the advanced stage. Although multiple therapeutic agents have been proven to be curative in ccRCC, their clinical application was limited due to the lack of reliable biomarkers. Considering the important role of basement membrane (BM) in tumor metastasis and TME regulation, we investigated the expression of BM-related genes in ccRCC and identified prognostic BM genes through differentially expression analysis and univariate cox regression analysis. Then, BM-related ccRCC subtypes were recognized through consensus non-negative matrix factorization based on the prognostic BM genes and evaluated with regard to clinical and TME features. Next, utilizing the differentially expressed genes between the BM-related subtypes, a risk scoring system BMRS was established after serial analysis of univariate cox regression analysis, lasso regression analysis, and multivariate cox regression analysis. Time-dependent ROC curve revealed the satisfactory prognosis predictive capacity of BMRS with internal, and external validation. Multivariate analysis proved the independent predictive ability of BMRS and a BMRS-based nomogram was constructed for clinical application. Some featured mutants were discovered through genomic analysis of the BMRS risk groups. Meanwhile, the BMRS groups were found to have distinct immune scores, immune cell infiltration levels, and immune-related functions. Moreover, with the help of data from The Cancer Immunome Atlas (TCIA) and Genomics of Drug Sensitivity in Cancer (GDSC), the potential of BMRS in predicting therapeutic response was evaluated and some possible therapeutic compounds were proposed through ConnectivityMap (CMap). For the practicability of BMRS, we validated the expression of BMRS-related genes in clinical samples. After all, we identified BM-related ccRCC subtypes with distinct clinical and TME features and constructed a risk scoring system for the prediction of prognosis, therapeutic responses, and potential therapeutic agents of ccRCC. As ccRCC systemic therapy continues to evolve, the risk scoring system BMRS we reported may assist in individualized medication administration.

## 1 Introduction

Globally, more than 430,000 individuals suffering from kidney cancers were newly diagnosed in 2020 and approximately 180,000 people died from this type of cancer (Sung et al., 2021). As one of the major subtypes, clear cell renal cell carcinoma (ccRCC) could earn a good prognosis when treated at an early stage. However, around one-third of ccRCC patients were found to be in the metastatic stage, requiring systemic therapy other than radical surgery (Jonasch et al., 2014). Although novel treatments including immunotherapies and targeted therapies were demonstrated to be curative in this chemoresistant cancer type, their clinical effects were uncontrollable due to the lack of predictive biomarkers for the therapeutic response and adverse events (Jhaveri and Perazella, 2018; Motzer et al., 2020a). Besides, it was demonstrated that combined therapy of immune checkpoint inhibitors (ICIs) and vascular endothelial growth factor (VEGF) tyrosine kinase inhibitors (TKIs) could exert better curative effects than monotherapy, leading to a requirement for more individualized markers for treatment selection (Amin and Hammers, 2018). Therefore, reliable predictive biomarkers ought to be developed for the prognosis and therapeutic response of ccRCC.

The tumor microenvironment (TME) is a complex ecosystem including immune cells, stromal cells, and extracellular matrix, surrounding and interacting with tumor cells (Blankenstein et al., 2012). As a highly immune-infiltrated cancer type, ccRCC cells were able to modulate the TME including immune cells for evasion of anti-cancer immunity through multiple mechanisms (Díaz-Montero et al., 2020). The understanding of these mechanisms could facilitate the application of cancer-specific therapies, such as ICIs, which restored anti-cancer immunity through interrupting the suppressive signals from the ccRCC cells (Motzer et al., 2015). Besides, accumulating evidence indicated that molecular classification of ccRCC into groups with distinct TME features could distinguish their prognosis and therapeutic response (de Velasco et al., 2017). Thus, it would be valuable to investigate the TME in ccRCC for the discovery of novel predictive biomarkers.

Basement membrane (BM) is a thin sheet of extracellular matrix (ECM) lining beneath endothelial and epithelial tissues, mainly composed of collagen IV and laminin (Yurchenco, 2011). It serves as one of the barriers preventing cancer cells from invasion, but its remodeling and stiffness would contribute to the metastasis of tumor (Chang and Chaudhuri, 2019). Some BM-related genes were revealed to be associated with the prognosis of RCC. Wragg et al. (2016) demonstrated that the high expression of LAMA4, a laminin component, was correlated with the poor prognosis of RCC (Wragg et al., 2016; Ho et al., 2017). Moreover, BM could mediate the signal transduction between the microenvironment and cells. As a major component of BM, laminin was revealed to have the ability to modulate the migration, activation and functionality of T lymphocytes within tumors (Liu et al., 2022). With these concerns, investigating the BM-related genes in ccRCC may assist in understanding the relationship between ccRCC and TME and developing predictive biomarkers.

**FIGURE 1:**
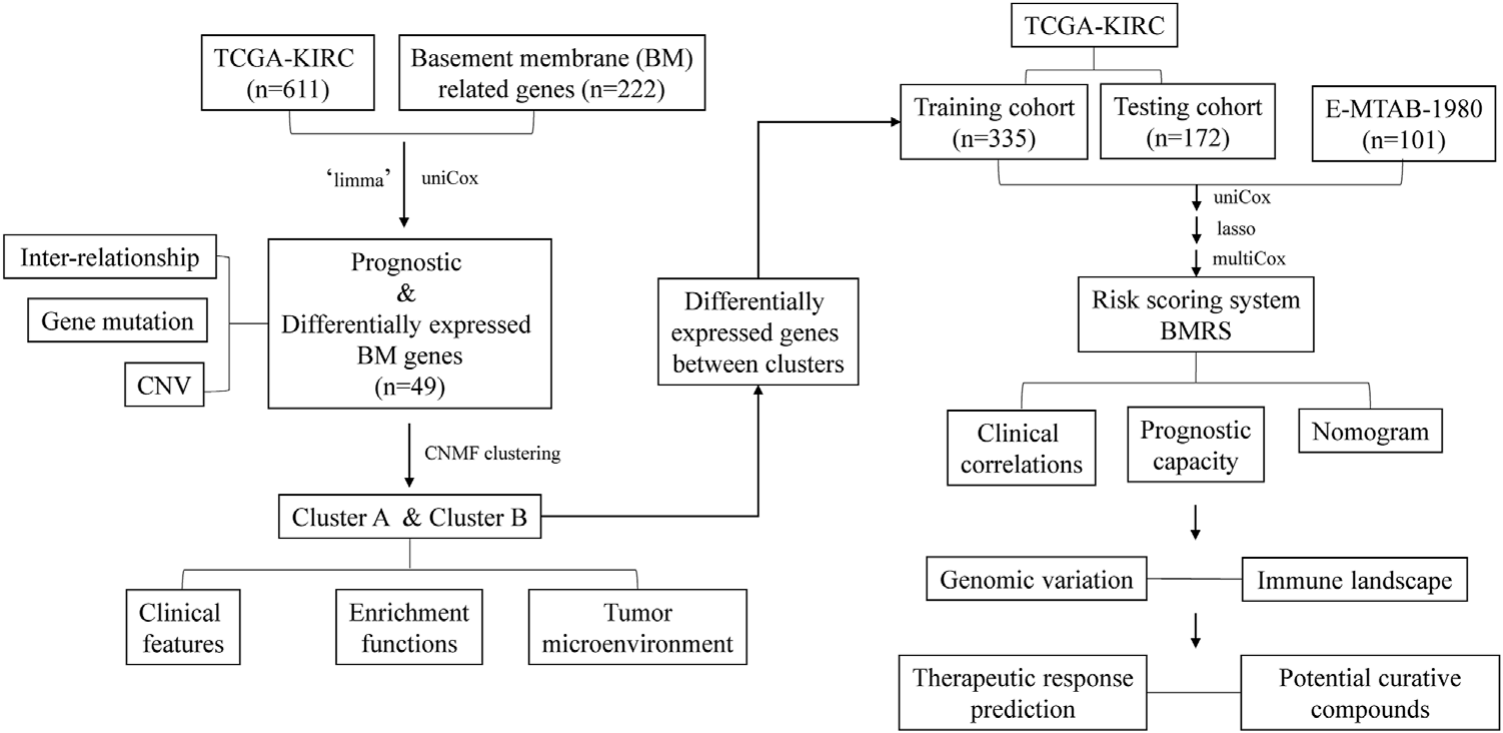
The flow chart of the current study.

In the current study (Figure 1), we investigated the expression of BM-related genes in ccRCC and used them to classify ccRCC patients into distinct subtypes, based on which a risk scoring system, BMRS, was established. Comprehensive analyses were conducted to evaluate the capacity of BMRS in distinguishing the prognosis, TME features, and therapeutic response of ccRCC. In this way, we constructed a gene-based BMRS for prognosis and treatment prediction of ccRCC and provided molecular candidates as novel therapeutic targets.

## 2 Materials and methods

### 2.1 Data acquisition

A ccRCC cohort including 539 tumor samples and 72 normal samples from the KIRC project of The Cancer Genome Atlas (TCGA) was selected and its RNA expression data, somatic mutation data, and the corresponding clinical data were extracted from the Genomic Data Commons Data Portal (https://portal.gdc.cancer.gov). ArrayExpress (https://www.ebi.ac.uk/arrayexpress) is another public database containing high-throughput genomic data from more than 75,000 experiments. Gene expression data of a ccRCC cohort, E-MTAB-1980, with 101 tumor samples were acquired from ArrayExpress and the survival information was obtained from previous research (Li et al., 2018). The expression data of both cohorts were transformed into a data format of transcripts per million (TPM) for better analysis. All the data were publicly available and no ethical consent was required.

### 2.2 Investigation of the basement membrane genes in ccRCC

Jayadev et al. discovered 222 protein-coding genes that were related to BM and human health (Jayadev et al., 2022). The expression of these genes was extracted from the TCGA cohort and differentially analyzed between tumor and normal samples with the help of the R package “limma” to identify the differentially expressed BM genes (Ritchie et al., 2015). Thereafter, univariate cox regression analysis facilitated the selection of the prognostic BM genes from those differentially expressed genes. For a better understanding of the expression of the prognostic BM genes in ccRCC, correlation analyses were performed to reveal their interrelationship. Besides, utilizing the R package “maftool” (Mayakonda et al., 2018), the variation in these genes was depicted including both somatic mutation status and copy number variations (CNV).

### 2.3 Discovery and investigation of basement membrane-related clusters in ccRCC

Consensus non-negative matrix factorization (CNMF) is a powerful method for the dimension reduction of genomic data to discover distinguished molecular patterns. Through the R package ‘CancerSubtypes’ (Xu et al., 2017), the expression data of the prognostic BM genes were used to classify the ccRCC samples in the TCGA cohort into 2, 3, and 4 clusters. Silhouette width is a measurement for the evaluation of the classifications and a larger average silhouette width means more valuable subtypes. Clusters with the highest average silhouette width were selected for the following analyses. To further investigate the value of these BM-related clusters, Kaplan-Meier survival analysis and chi-square test were conducted to visualize the clinical difference between them. Meanwhile, though GSEA 4.1.0, gene sets enrichment analysis (GSEA) together with gene ontology (GO) gene sets assisted the understanding of the molecular functions enriched in each cluster. Furthermore, Estimation of STromal and Immune cells in MAlignant Tumor tissues using Expression data (ESTIMATE) (Yoshihara et al., 2013) was utilized to quantify the immune and stromal status of ccRCC samples and the resulting scores were compared between each cluster to discover whether there were differences in their TME. Besides, we calculated the infiltrating levels of 23 tumor infiltrating immune cells (TIIC) through single sample GSEA (ssGSEA) and 14 stoma cells by xCell (https://xcell.ucsf.edu/). By comparing the infiltrating level of these cells between different clusters, their TME differences could be better understood.

### 2.4 Construction of a gene expression-based risk score system

After discovering the distinct features between the BM-related subtypes, we tended to construct a risk score system based on these clusters. First, the differentially expressed genes between the clusters were identified. Then, the TCGA cohort was randomly divided into a training cohort and a testing cohort in a ratio of 7 to 3. In the training cohort, expression of the differentially expressed genes was extracted and used for univariate cox regression analysis to select the prognostic genes. Through R package ‘glmnet’ (Friedman et al., 2010), Least Absolute Selection and Shrinkage Operator (LASSO) regression analysis facilitated identifying those genes with higher association with ccRCC prognosis, and multivariate cox regression analysis was utilized to choose the best genes for construction of the BM-related risk score system (BMRS): risk score = coefficient 1*gene expression 1 + … + coefficient n * gene expression n (1 to n represent each prognostic genes).

### 2.5 Analysis of the prognostic predictive capacity of BMRS

To investigate the predictive ability of BMRS, the TCGA training cohort, TCGA testing cohort, TCGA cohort, and E-MTAB-1980 cohort were respectively divided into high and low risk groups according to the median risk scores. Kaplan-Meier analysis was applied in each pair of high and low risk groups to reveal the relationship between risk score and the overall survival (OS) of ccRCC patients. Subsequently, with the help of time-dependent Receiver Operating Characteristic (tROC) curves, the predictive power of the risk scoring system for the 1-, 3-, and 5-year OS of each cohort were illustrated and the corresponding values of area under curve (AUC) were calculated. Additionally, the clinical features (age, gender, Fuhrmann grade, and AJCC stage) in high and low risk groups of the TCGA cohort were compared through chi-square test. Meanwhile, the ability of BMRS in differentiating ccRCC OS was tested under different clinical statuses by Kaplan-Meier survival analysis.

### 2.6 Establishment of a clinical predictive nomogram

For better application in the clinic, BMRS (high risk *vs*. low risk) combined with age (<65-year-old *vs*. >=65-year-old), gender (female *vs*. male), Fuhrmann grade (Grade 1/2 *vs*. Grade 3/4), AJCC stage (Stage I/II *vs*. Stage III/IV), T stage (T1/2 *vs*. T3/4), N stage (N0 *vs*. N1), and M stage (M0 *vs*. M1) were incorporated into univariate and multivariate cox regression analyses to demonstrate its predictive value. Furthermore, the resulting values of the multivariate cox regression analysis were used to construct a nomogram for the prediction of ccRCC survival. The predictive capability of the nomogram for 3-and 5-year survival was assessed by calibration plot. Besides, decision curve analysis (DCA) was utilized to compare the net benefit of BMRS, AJCC stage and two previous BM-related gene signatures (Zhou et al., 2022a; Xiong et al., 2022) in predicting 3- and 5-year survival of ccRCC. TCGA cohort was separated into high and low risk groups according to the median of nomogram scores and was analyzed to identify their survival difference.

### 2.7 Investigation of the genomic variation in different BMRS groups

The genomic features in high and low BMRS groups were illustrated and compared through the R package ‘maftool’. The variation status of the top 20 mutation genes in both groups was depicted and all the mutated genes were compared between groups to identify the group-specific mutation. At the same time, by applying pairwise Fisher’s exact test between every two genes, we wanted to discover whether there were some exclusive or co-occurrence gene pairs in the high BMRS group. Furthermore, some tumor heterogeneity-related features, including single nucleotide variation (SNV), homologous recombination defects (HRD), cancer testis antigen (CTA), and intratumor heterogeneity (ITH) were introduced from previous research (Thorsson et al., 2018) and compared between different BMRS groups.

### 2.8 Analysis of the immune landscape related to BMRS

In order to investigate the BMRS-related biological processes, cellular components, and molecular functions, the genes that were differentially upregulated in the high BMRS group were chosen and incorporated into GO functional analysis through the R package ‘clusterProfiler’ (Wu et al., 2021). Concerning the immune microenvironment in ccRCC, the immune scores calculated from ESTIMATE and the infiltrating scores of 23 TIICs were compared between BMRS groups. Each TIIC was analyzed through Kaplan-Meier survival analysis to discover its relationship with ccRCC prognosis. Meanwhile, ssGSEA algorithm was utilized to induce scores representing immune suppression and some immune-related functions with the help of previously published gene signatures (Yi et al., 2020). In addition, a publicly accessible website called Tumor Immune Dysfunction and Exclusion (TIDE, http://tide.dfci.harvard.edu) which provides a platform for estimation of T cells dysfunction scores based on gene expression data, was adopted to compare the T cells status between high and low BMRS clusters.

### 2.9 Exploration of the therapeutic predictive potential of BMRS

The gene expression of several popular inhibitory immune checkpoints was extracted and differentially analyzed between high and low BMRS groups. Additionally, the immunophenoscores, which are estimated scores of immunotherapeutic response to ICIs for TCGA samples, were obtained from The Cancer Immunome Atlas (TCIA, https://tcia.at/home) and compared between BMRS groups. Except for immunotherapeutic response, sensitivity toward some therapeutic drugs (Axitinib, Pazopanib, Sorafenib, Sunitinib, and Mitomycin C) clinically used for ccRCC patients were predicted through R package ‘pRRophetic’ (Barbour et al., 2014), which connects a large amount of gene expression and drug sensitivity data in Genomics of Drug Sensitivity in Cancer (GDSC, https://www.cancerrxgene.org). Furthermore, the gene expression of immune chemokines, immune receptors, and major histocompatibility complex (MHC) molecules was correlated with BMRS with the concern of discovering potential therapeutic targets. Besides, we uploaded the top 150 upregulated genes in high and low BMRS groups respectively to ConnectivityMap (CMap, https://clue.io) for exploration of potential therapeutic compounds for patients in each group.

### 2.10 Transcriptome sequencing analysis

To further prove the applicability of BMRS, we collected clinical ccRCC and adjacent normal samples (18 ccRCC and 6 adjacent normal samples) for transcriptome sequencing analysis. This project was supported by the hospital ethics committee and consent was acquired from all the patients. In line with the protocol of the manufacturer, each ccRCC and adjacent normal samples underwent paired-end sequencing on the NovaSeq 6000 high-throughput sequencing platform (Illumina, United States) to remove sequencing reads containing aptamer sequences and low-quality reads as well as bases. Then, high-quality pairwise reads were aligned to the human genome GRCh38 through HISAT2 (v2.1.1), generating BAM files. BAM files were arranged by samtools (v1.15.1) and then counted with the help of Subread (v2.0.1). Raw counts of transcripts per gene were converted to the format of TPM, allowing better analysis of gene expression between samples. Thereafter, the genes enrolled in BMRS were differentially analyzed between the normal and ccRCC samples.

### 2.11 Statistical analysis

All the analyses process in the current study were achieved through the usage of R 4.1.0 and R studio Desktop 2022.07.1 + 554. The graphs displayed were drawn by R studio Desktop 2022.07.1 + 554 and Adobe Illustrator CS6 (64 Bit). During differentially expression analysis, genes with the absolute value of log fold change (logFC) more than were selected. *p*-value less than 0.05 was regarded as significant for all analyses.

## 3 Results

### 3.1 Identification of the differentially expressed and prognostic BM genes

**FIGURE 2:**
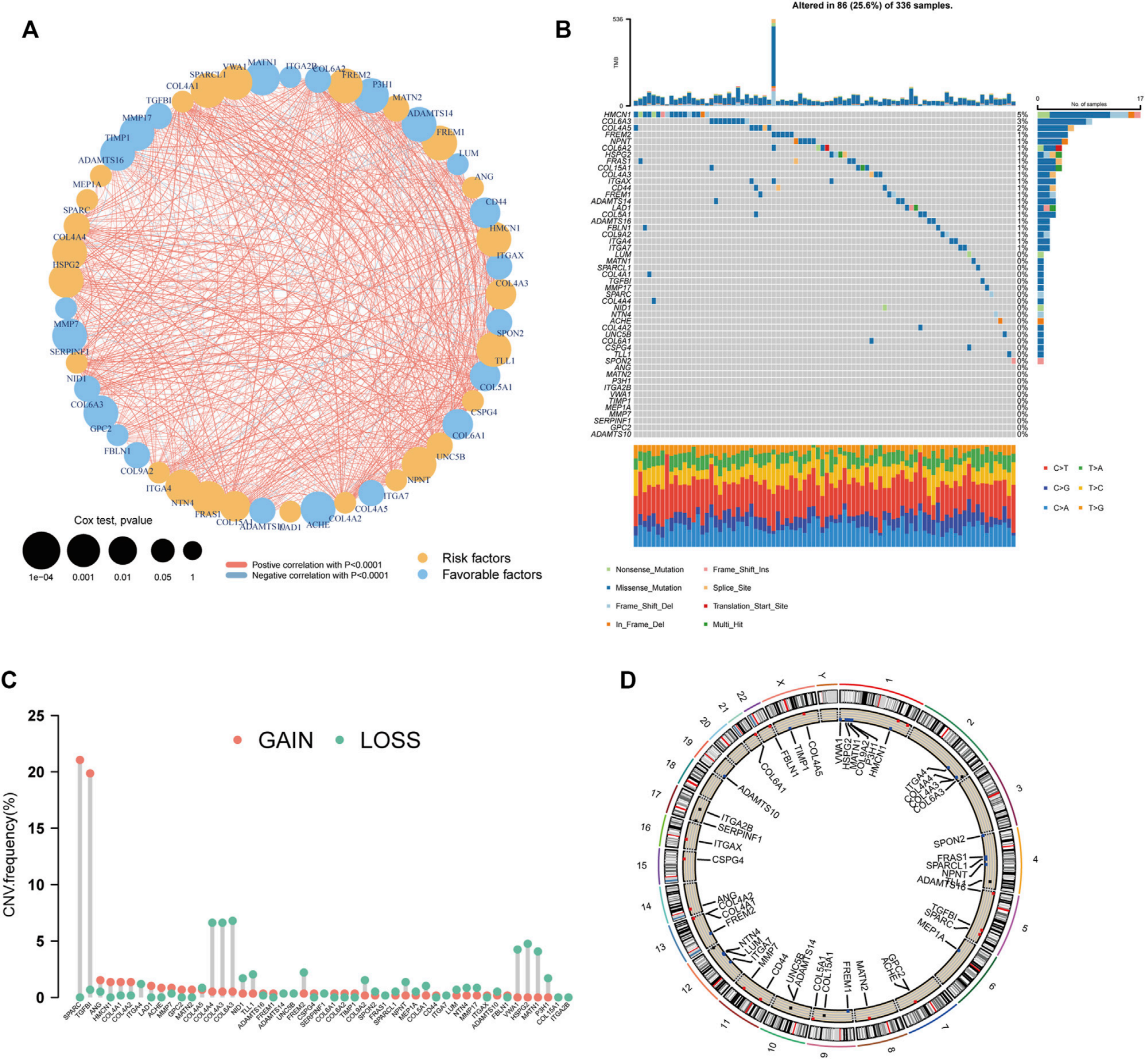
Genetic analyses of the BM-related genes. **(A)** The inter-relationship between each BM-related gene and the role of these genes in the prognosis of ccRCC. **(B)** The waterfall plot depicting the mutation of the prognostic BM genes. **(C)** The CNV frequency of the prognostic BM genes. **(D)** The distribution of the prognostic BM genes in each chromosome with CNV gain colored red, CNV loss colored blue and no CNV colored black.

Differential expression analysis of the 222 BM genes between 539 ccRCC samples and 72 normal samples revealed that there were 106 differentially expressed BM genes, in which 39 genes were downregulated and 67 genes were upregulated (Supplementary Table S1). Then, after univariate cox regression analysis, 49 BM genes were demonstrated to be prognostic including 26 protective genes and 23 risk genes (Supplementary Table S2). A substantial positive correlation existed between these prognostic BM genes indicating that they were highly interconnected (Figure 2A). As for the genomic variation that happened in these genes, 25.6% of the ccRCC samples possessed prognostic BM gene alterations and more of these alterations were missense mutations (Figure 2B). The top 3 mutated genes were HMCN1 (5%, risk gene), COL6A3 (3%, protective gene), and COL4A5 (2%, protective gene). In addition, the CNV of these genes was analyzed and a relatively low frequency of CNV was discovered, except for SPARC (21.0%, gain of function), and TGFBI (19.8%, gain of function) (Figures 2C, D).

### 3.2 Recognition of BM-based clusters of ccRCC

**FIGURE 3:**
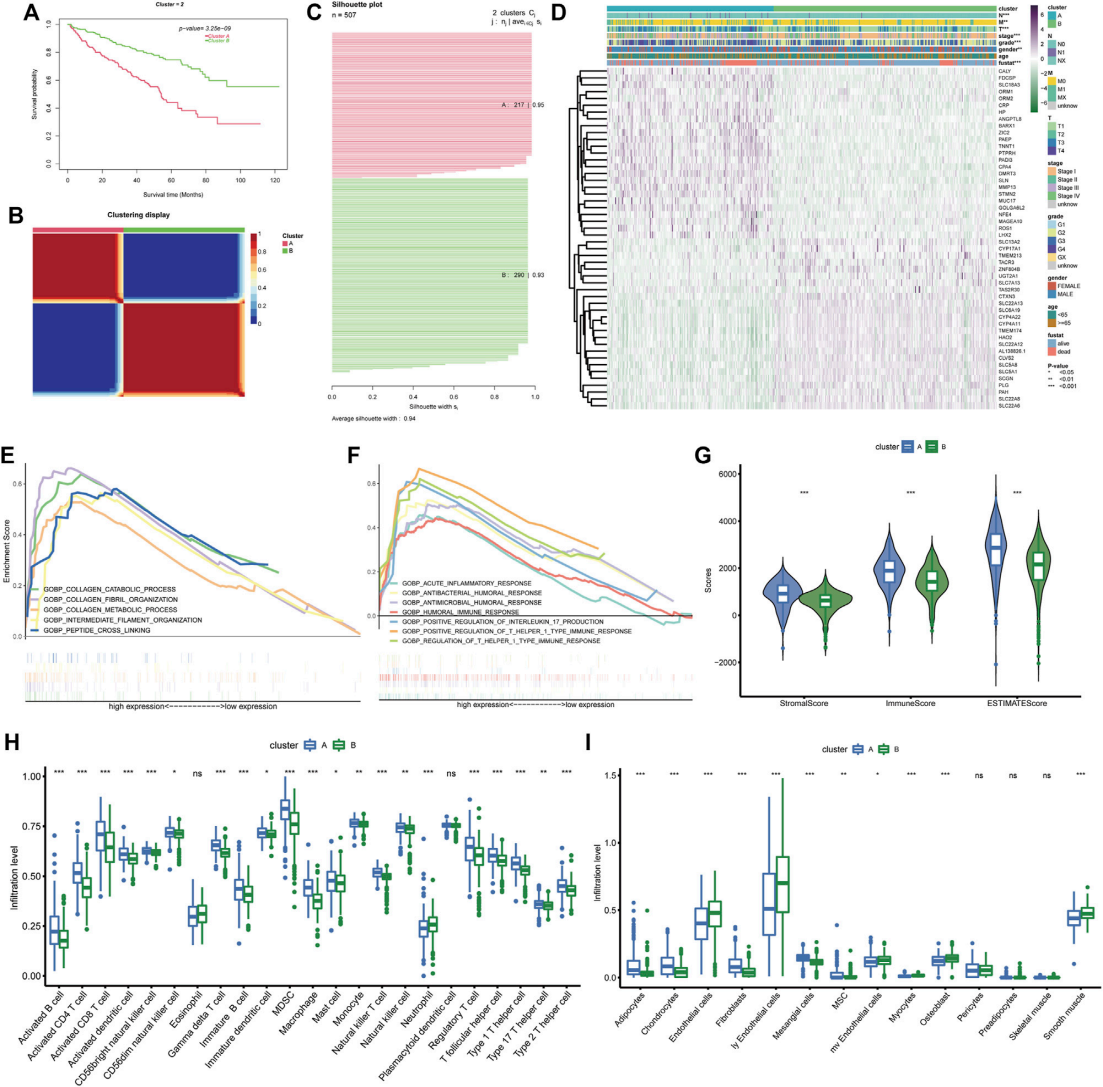
BM gene-based classification of ccRCC patients. **(A)** Survival analysis of the two ccRCC clusters classified based on BM genes. **(B)** The sample similarity matrix plot of the two identified clusters. **(C)** The Silhouette width plot of the two BM-related subtypes. **(D)** The heatmap depicting the distinct clinical features and gene expression between cluster **(A)** and cluster **(B)**. **(E, F)**. The biological processes that enriched in cluster **(A)**. **(G)** Comparison of the stromal score, immune score and ESTIMATE score between cluster **(A)** and cluster **(B)**. **(H, I)** The different infiltration levels of immune cells and stromal cells between the two BM-related subtypes.

The 49 differentially expressed and prognostic BM genes were further used for the classification of ccRCC patients through CNMF algorithm. As the results show, the samples could be classified into two separate clusters A and B, in which cluster A had significantly lower OS than cluster B and the average silhouette width was 0.94 (Figures 3A–C). For the reason that the average silhouette widths were relatively low, classifications of 3, 4, and 5 clusters were not under consideration (Supplementary Figure S1). By comparing the clinical features in both clusters, we discovered that the ccRCC samples in cluster A had more advanced features including Fuhrmann grade, AJCC stage, T stage, N stage, and M stage. There were more males in cluster A than in cluster B while the age distributions in both clusters were similar (Figure 3D). The differentially expressed analysis also indicated that some metastasis-related genes, such as MMP13 and ROS1 were upregulated in cluster A. The following GSEA analysis demonstrated that basement membrane-related functions like collagen fibril organization and collagen catabolic process were more prominent in cluster A. Meanwhile, some immune-related biological processes were notably enriched in cluster A, such as positive regulation of T helper 1 type immune response and positive regulation of interleukin 17 production (Figures 3E, F). Considering these enriched immune functions, we further investigated the microenvironment components in both clusters through ESTIMATE and ssGSEA. It was demonstrated that the stromal score, immune score and ESTIMATE score in cluster A were higher than those in cluster B, indicating the rich stromal, immune components and low tumor purity in cluster A (Figure 3G). At the same time, cluster A had a higher level of nearly every type of infiltrating immune cell than cluster B except for eosinophils, neutrophils, and plasmacytoid dendritic cell (Figure 3H). As for stromal cells, more adipocytes, chondrocytes, fibroblasts, and mesangial cells were found in cluster A while cluster B possessed more endothelial cells, lymphatic endothelial cells and microvascular endothelial cells (Figure 3I). Therefore, the prognostic BM genes could divide ccRCC samples into two clusters with distinct clinical and TME features.

### 3.3 Construction of BMRS risk scoring system for prognosis prediction

**FIGURE 4:**
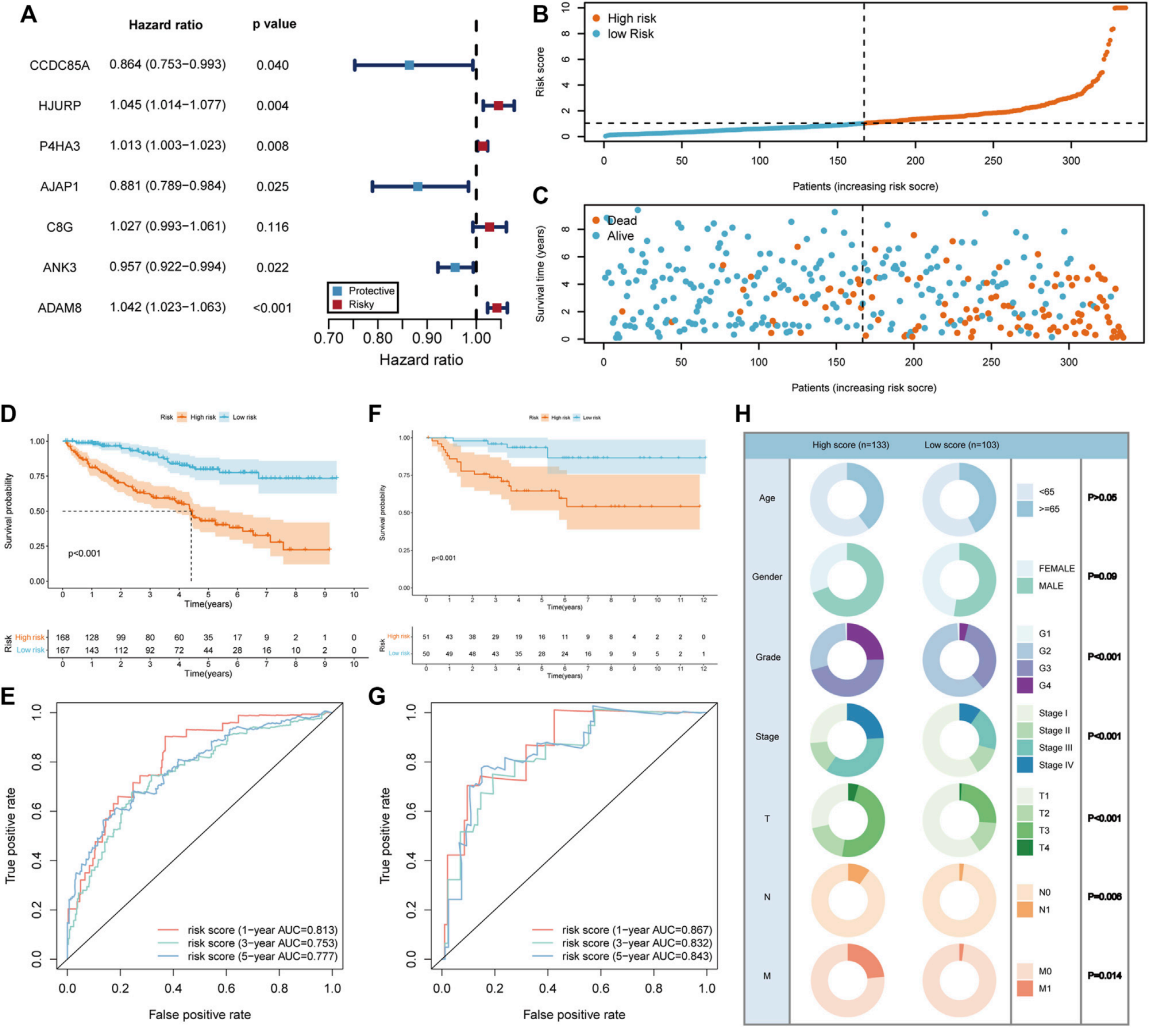
Construction and evaluation of the risk scoring system BMRS. **(A)** The forest plot illustrating the 7 genes resulted from multivariate cox regression analysis. **(B, C)** Distribution of the risk scores and survival status of ccRCC patients in the TCGA training cohort. **(D, F)** The Kaplan Meier survival curve comparing the overall survival between high and low risk groups in the TCGA training cohort and E-MTAB-1980 cohort respectively. **(E, G)** The tROC curve of the risk score for 1-, 3-, and 5-year survival in the TCGA training cohort and E-MTAB-1980 cohort. **(H)** Comparison of the clinical features in high and low risk groups.

Since the BM gene-based ccRCC subtypes could discriminate prognostic and clinical features, we would like to derive a more applicable risk scoring system from these subtypes. In the TCGA training cohort, the differentially expressed genes between clusters A and B were identified (Supplementary Figure S2A). Based on these genes, univariate cox regression analysis was conducted and induced 414 prognostic genes (Supplementary Table S3). Subsequently, LASSO algorithm was applied to obtain 12 genes that were significantly associated with the prognosis of ccRCC (Supplementary Figure S2B, C). Then, these 12 genes were incorporated into multivariate cox regression analysis (Figure 4A), inducing a risk scoring system, BMRS, based on 7 genes, CCDC85A, AJAP1, ANK3, P4HA3, C8G, ADAM8, HJURP.

### 3.4 Evaluation of the predictive ability of BMRS

For the evaluation of BMRS system, we calculated risk scores for each sample in TCGA training cohort and divided them into high and low risk groups according to the median risk scores (Figure 4B). It could be recognized that samples with high risk were more frequently dead than those with low risk (Figure 4C). At the same time, survival analysis demonstrated that the high risk group had longer OS than the low risk group (Figure 4D). The results of tROC curve proved that BMRS system possessed a great prognostic capacity for 1-year (AUC = 0.813), 3-year (AUC = 0.753), and 5-year (AUC = 0.777) survival (Figure 4E). The same analyses were applied in the TCGA testing cohort and TCGA cohort as an internal validation and delivered similar results (Supplementary Figure S2D–K). As an external validation, the survival analysis result and tROC curve derived from E-MTAB-1980 also supported that BMRS could discriminate the prognosis of ccRCC and had high accuracy (Figures 4F, G, 1-year AUC = 0.867, 3-year AUC = 0.832, and 5-year AUC = 0.843). Furthermore, survival analyses of high and low risk groups in the TCGA cohort under various clinical situations were conducted and revealed that BMRS could discriminate ccRCC survival in most situations (Supplementary Figure S3). Meanwhile, the Fuhrmann grade, AJCC stage, T stage, N stage, and M stage in the high risk group were notably higher than those in the low risk group, which was in line with the poor prognosis in the high risk group (Figure 4H). Moreover, we evaluated the gene expression of the genes used for BMRS and the results demonstrated that most of the genes were differentially expressed in ccRCC (Supplementary Table S4), consistent with the above analysis. In this way, a reliable risk scoring system BMRS was constructed with considerable predictive capacity.

### 3.5 Establishment of a clinical nomogram based on BMRS

**FIGURE 5:**
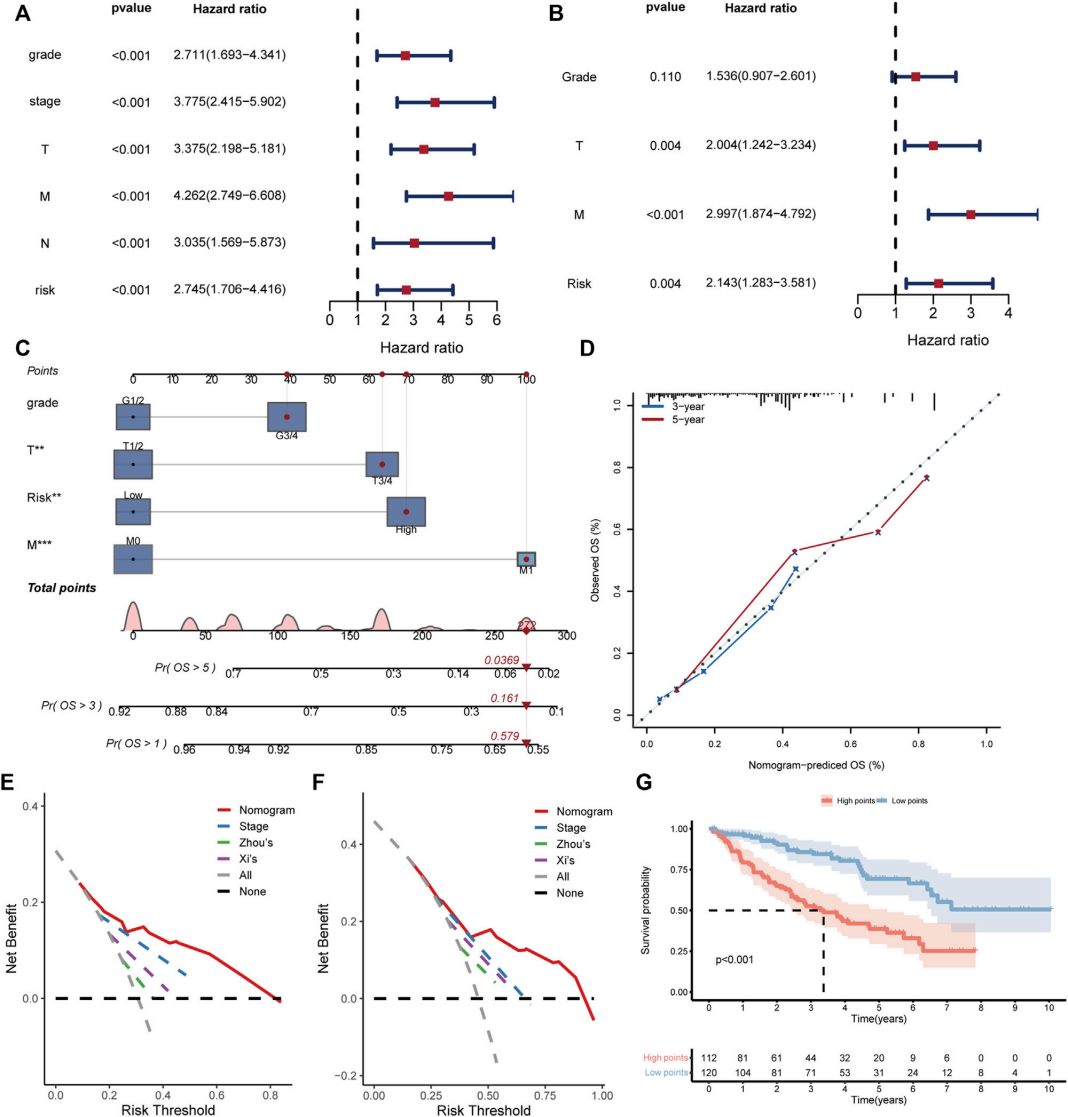
Establishment and evaluation of the clinical predictive nomogram. **(A, B)** The forest plots depicting the results of univariate cox regression analysis and multivariate cox regression analysis respectively. **(C)** The nomogram based on grade, T stage, M stage, and risk score. The red dot lines represented an example of the total point calculation. **(D)** The calibration curve testing predictability of the nomogram for 3- and 5-year survival. **(E, F)** The DCA curves comparing the net benefit of nomogram, TNM stage, Zhou’s and Xi’s gene signatures for prediction of 3- and 5-year survival respectively. **(G)** The Kaplan Meier survival curve comparing the overall survival of ccRCC patients with high and low points.

For better application of the BMRS in clinic, we evaluated its predictive ability with the consideration of the clinical variables. Analyzing risk score and clinical variables cooperatively, univariate cox regression analysis revealed that only age and gender could not predict ccRCC prognosis (Figure 5A). The following multivariate cox regression analysis uncovered the independent prognostic predictive ability of BMRS, T stage and M stage (Figure 5B). Thereafter, the Fuhrmann grade, T stage, M stage and BMRS risk were incorporated to establish a nomogram (Figure 5C). The calibration curve depicted the 3-year and 5-year OS predicted by the nomogram had a satisfactory consistency with those observed in clinic (Figure 5D). While compared with the clinically popular TNM stage, Zhou’s and Xi’s gene signatures, the nomogram could deliver higher net benefit in 3-year (Figure 5E) and 5-year OS prediction (Figure 5F). The survival analysis also demonstrated that samples with high points had significantly lower OS than those with low points (Figure 5G). Thus, BMRS could not only independently predict ccRCC survival but also assist in establishing more competitive predictive methods.

### 3.6 The genomic variation in different risk groups

**FIGURE 6:**
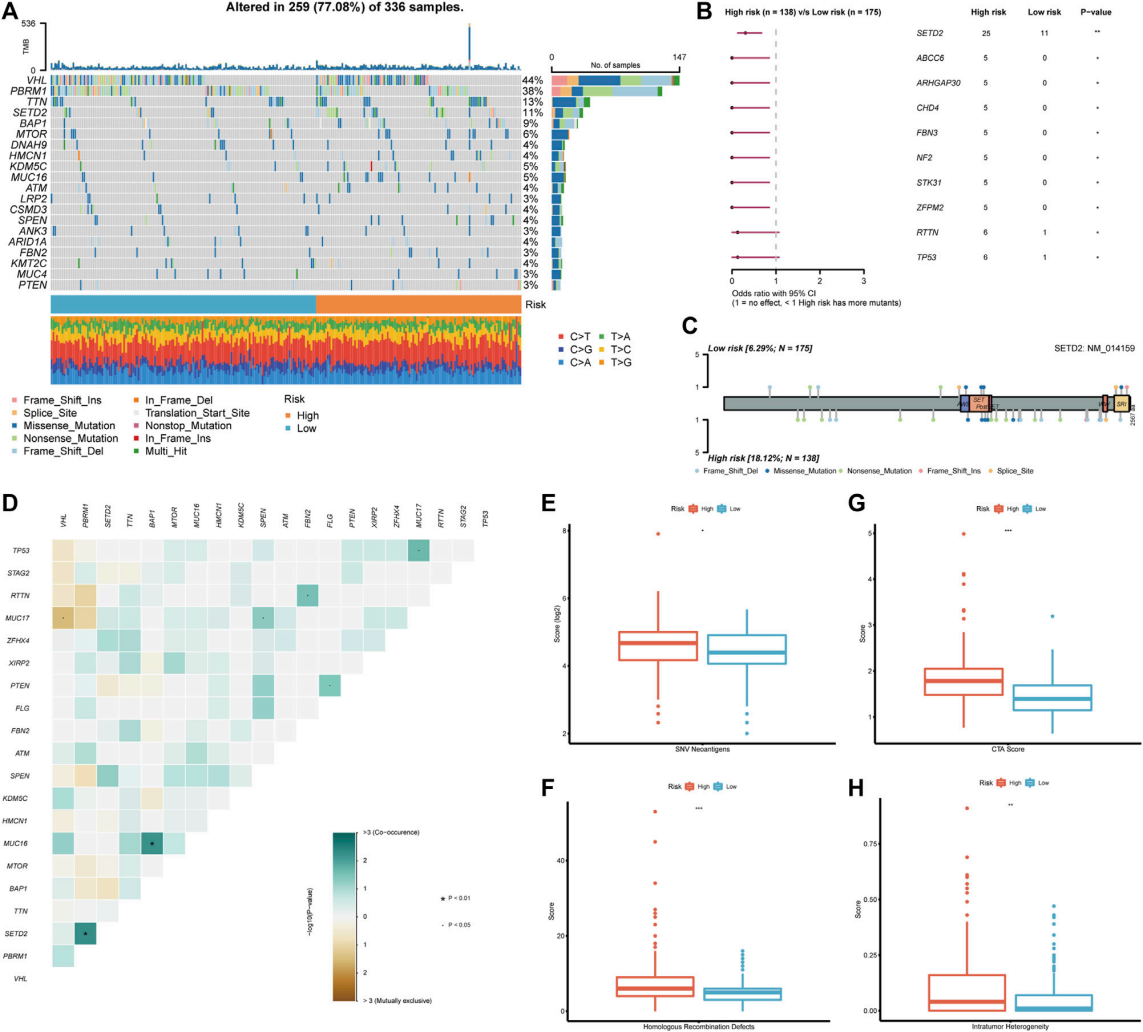
Genomic analysis in accordance with the BMRS risk groups. **(A)** The waterfall plot representing the top 20 mutated genes in both risk groups. **(B)** The mutants that significantly differentiated between high and low risk groups. **(C)** Exhibition of the mutation sites and types of SETD2 in both risk groups. **(D)** The correlation of the mutants in the high risk group. **(E–H)** Comparison of SNV neoantigens, HRD, CTA score, and ITH between high and low risk groups.

After discovering the clinical significance of BMRS, further analyses of the underlying genomic alterations were under concern. In both risk groups, the mutation of VHL (44%), PBRM1 (38%), TTN (13%), and SETD2 (11%) comprised the major part of all gene alterations (Figure 6A). The following comparison revealed that 10 mutants were occurring more frequently in the high risk group (Figure 6B). Among these mutants, SETD2 was the most frequent and significant one and most of its mutation happened in the non-coding area (Figure 6C). Besides, the discovery of the relationship between gene variations uncovered a notable co-occurance of SETD2 and PBRM1 mutation. At the same time, the mutation of MUC16 and BAP1 also exhibited a significant positive inter-relationship while MUC17 and VHL mutation were mutually exclusive (Figure 6D). Apart from these mutational differences, the high risk group was demonstrated to have higher scores of SNV antigens and HRD, indicating the high genomic heterogeneity in patients with high BMRS risk (Figures 6E, F). Meanwhile, the CTA and ITH scores in the high risk group were significantly higher than those in the low risk group (Figures 6G, H).

### 3.7 The association between BMRS and the immune landscape of ccRCC

**FIGURE 7:**
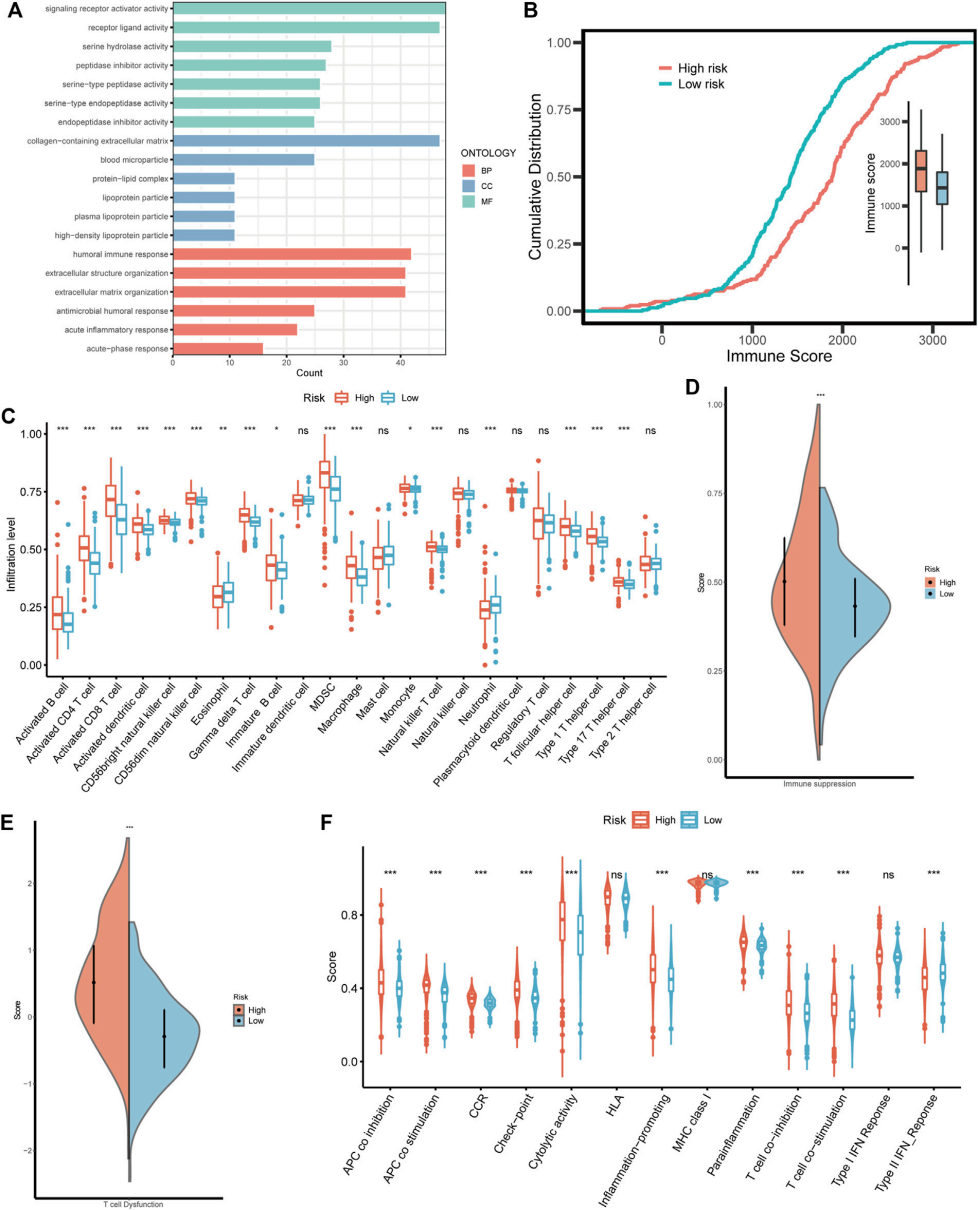
The immune landscape in different BMRS risk groups. **(A)** The enriched GO functions in the high risk group. **(B)** The different cumulative distributions of the immune score in different risk groups. **(C)** The distinct infiltration level of the immune cells in high and low risk groups. **(D, E)** Comparison of the scores of immune suppression and T cell dysfunction between the two BMRS groups. **(F)** The difference in immune functions between high and low risk groups.

Considering that BMRS was derived from the BM-related clusters with distinct functional and immunal features, we also investigated the functions associated with BMRS. Similar to the above results, the high risk group was enriched with BM-related functions, such as extracellular structure organization and extracellular matrix organization. Meanwhile, there were some immune involvements recognized in the high risk group including humoral immune response and acute inflammatory response (Figure 7A). The comparison of immune scores between the two risk groups also indicated that the high risk group had a higher immune level than the low risk group (Figure 7B). For a better understanding of this immune involvement, we further evaluated the infiltrating immune cells in each group. As the results showed, most of the immune cells were more frequently infiltrated in the high risk group than in the low risk group except for eosinophil and neutrophil whose infiltrating levels were lower in the high risk group (Figure 7C). Survival analyses were conducted for each immune cell and it was revealed that some innate immune cells like neutrophil, and mast cell were protective cells. Most of the immune cells enriched in the high risk group were associated with poor survival, including suppressive immune cells (e.g., MDSC) and effector immune cells (e.g., activated CD8 T cell). For an exploration of this connection between abundant immune infiltration and poor survival, the functionality of the immune environment was assessed, delivering a result that ccRCC samples with high risk scored higher on immune suppression and T cell dysfunction than those with low risk (Figures 7D, E). Moreover, although high-risk samples had a higher level of most immune functions like APC co-stimulation and T cell co-stimulation, their scores of some negative immune functions such as APC co-inhibition and T cell co-inhibition were also greater than low-risk samples (Figure 7F). Therefore, BMRS may have a relationship with the suppressive immune microenvironment of ccRCC.

### 3.8 The therapeutic predictive potential of BMRS

**FIGURE 8:**
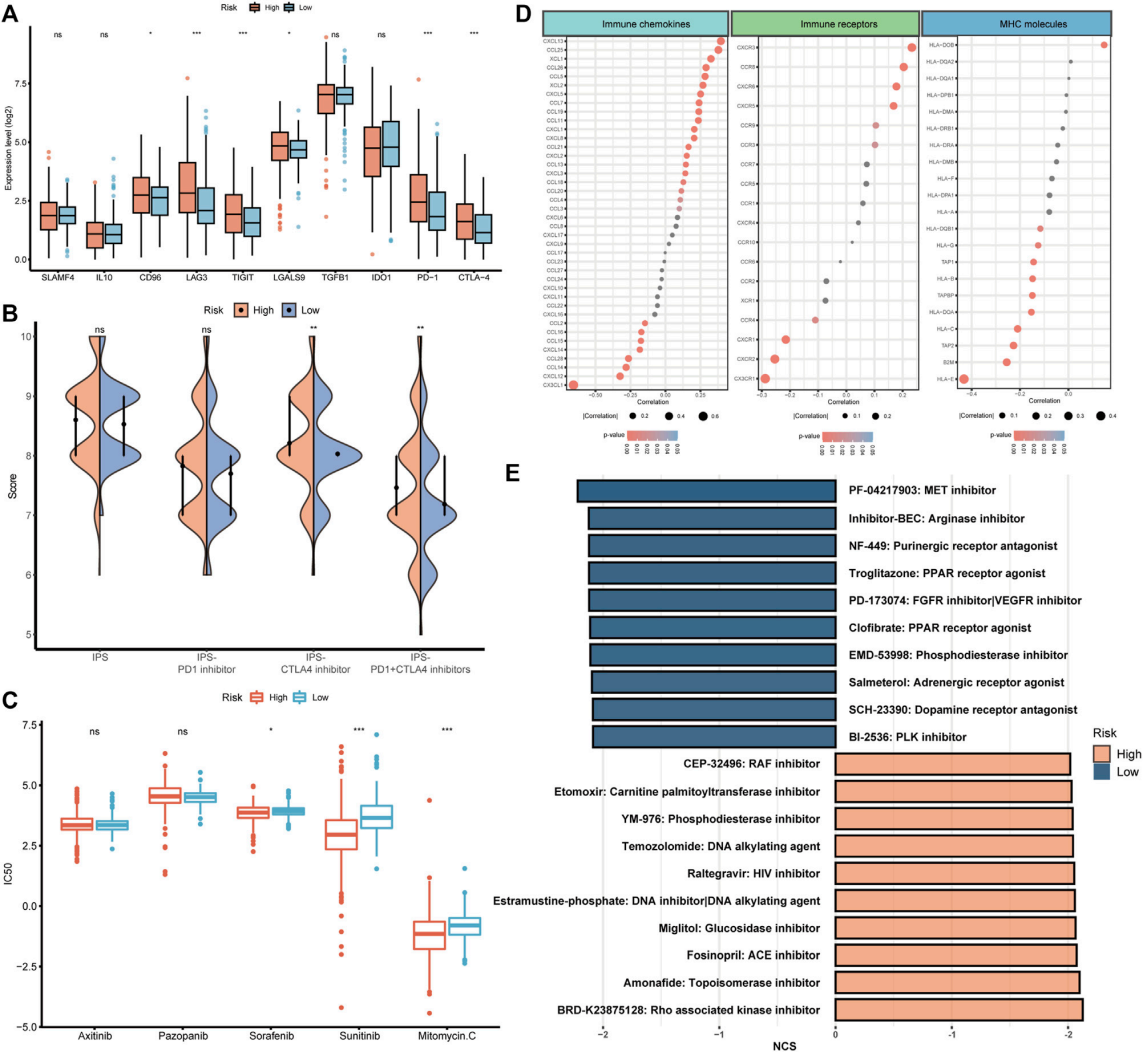
The potential of BMRS in guiding ccRCC treatment. **(A)** Comparison of the inhibitory immune checkpoint expression in different risk groups. **(B)** The IPS difference of4 types of ICI therapies in high and low BMRS groups. **(C)** Differential analysis of the IC50 of 5 therapeutic agents between BMRS risk groups. **(D)** Correlation between BMRS risk scores and the expression level of immune checkpoints, immune receptors and MHC molecules. **(E)** Potential therapeutic compounds predicted for high and low risk groups respectively.

After the investigation of BMRS and the immune microenvironment, the potential relationship between BMRS and immunotherapies was under consideration. Differential analysis of the gene expression of inhibitory immune checkpoints showed that PD1, CTLA4, LAG3, and TIGIT were significantly upregulated in the high risk group (Figure 8A). Data on immunotherapeutic response predicted by TCIA demonstrated that high-risk ccRCC patients possessed a higher level of IPS to CTLA4 inhibitor and combination of PD1 and CTLA4 inhibitors than low-risk patients, indicating high-risk patients may respond better toward these two strategies of immunotherapies (Figure 8B). Apart from immunotherapies, we also correlated BMRS with some therapeutic drugs used in clinic. The half maximal inhibitory concentration (IC50) of sorafenib, sunitinib, and mitomycin C. were notably lower in the high risk group than in low risk group, suggesting that ccRCC patients with high risk may have better outcomes receiving these drugs (Figure 8C).

To discover some additional therapeutic targets, we analyzed the correlation between BMRS and some immune-related molecules (Figure 8D). There were multiple immune checkpoints (e.g., CXCL13, and CCL25), immune receptors (e.g., CXCR5, and CCR6) and MHC molecules (e.g., HLA-E) significantly related to BMRS, providing potential targets for therapeutic investigation. Moreover, as predicted by CMap (Figure 8E), molecular compounds like BRD-K23875128, which is Rho associated kinase inhibitor, could serve as therapeutic drugs for high-risk ccRCC patients. Some other compounds were also estimated to be effective in low-risk ccRCC patients, for example, PF-04217903, which is a kind of arginase inhibitor.

## 4 Discussion

With the progression of ccRCC research, more treatment options were available to improve the prognosis of ccRCC patients (Choueiri and Motzer, 2017). However, the existing predictive markers for the prognosis and therapeutic response of ccRCC had limited functions due to the heterogeneity of this type of patient (Greef and Eisen, 2016). As a component of ECM, BM serves as not only a physical barrier against tumor invasion and metastasis but also a mediator of signals between microenvironment and cells (Bissell and Hines, 2011), indicating its potential as a target for the investigation of novel biomarkers. Through analyses of the expression of BM-related genes, we discovered two subtypes with distinct clinical and microenvironmental features and established a 7-gene risk scoring system for the prediction of prognosis and treatment response of ccRCC patients.

According to statistics, the prognosis of ccRCC was largely influenced by the occurrence of distant metastasis, which could decrease the 5-year survival rate to less than 20% (Siegel et al., 2022). In line with this finding, our results demonstrated that BM-related genes could divide ccRCC patients into two clusters with distinct prognosis features. This could be explained by the defensive role of BM in preventing the tumor cells from invading the stroma (Valastyan and Weinberg, 2011). Matrix metalloproteinases (MMPs), a series of zinc-dependent proteinases, played an important role in the degradation of BM to weaken its barrier capacity. Without MMPs, the tumor cells could hardly squeeze through the nanosized pores in the BM (Wolf et al., 2013; Eatemadi et al., 2017). It was demonstrated that the increased expression of MMPs was correlated with the invasion and poor prognosis of carcinomas (Winer et al., 2018), and in our study, the upregulation of MMP13 was identified in cluster A. Moreover, the high infiltration of fibroblasts in cluster A could also be associated with the expression of MMPs. It was reported that tumor cells induced the secretion of MMPs from cancer-associated fibroblasts (CAFs) to modulate BM (Kessenbrock et al., 2010). Conversely, MMPs such as MMP1 secreted from tumor cells promote the transdifferentiation of fibroblasts into CAFs (Heneberg, 2016). In addition to the chemical changes, BM could also be modulated into an invasion-favored status. A recent study revealed that in those BM with high plasticity, cells could mechanically enlarge the nanosized pores and migrate through BM without the help of MMPs (Wisdom et al., 2018). Meanwhile, the stiffness of BM could influence tumor invasion through a protein called netrin-4 (Net4). Net4 in BM mechanistically bound to laminin and diluted laminin ternary node complex, softening the BM and making it more resistant to tumor cell invasion (Reuten et al., 2021). Therefore, the different statuses of BM could influence the prognosis of ccRCC and may facilitate the survival prediction.

Based on the BM-related clusters, we constructed a risk scoring system called BMRS. Genes included in BMRS were found to be correlated with the progression and metastasis of cancers including both risk and protective genes. Holliday junction recognition protein (HJURP) is a kind of centromeric protein, being essential for the stimulation of chromosome division and cell mitosis (Zhang et al., 2021). Its upregulation was correlated with an increased invasion and migration capacity of cancer (Chen et al., 2019). Prolyl 4-hydroxylase alpha subunit 3 (P4HA3) is also a risk gene that could strengthen the motility and invasiveness of tumors (Song et al., 2018). As an enzymic subunit of prolyl 4-hydroxylase, P4HA3 was critical for the stability of collagen and its dysregulation would directly activate the invasiveness potential of tumor cells (Nakasuka et al., 2021). Meanwhile, a disintegrin and metalloproteinase 8 (ADAM8) was another gene related to enzyme production and could promote tumor metastasis probably through the degradation of ECM components (Conrad et al., 2019). Apart from risk genes, BMRS also contained protective genes including adherens junctional associated protein-1 (AJAP1) and ankyrin G (ANK3). AJAP1 belonged to multi-protein complexes named adherens junction, which is critical for cell adhesion and growth inhibition (Xu et al., 2019; Zhou et al., 2022b). ANK3, a family member of ankyrins, assisted in maintaining cell stability through anchoring cytoskeleton to the cell membrane (Wang et al., 2016). The downregulation of both genes could lead to the proliferation and invasion of cancer cells. However, little was known about coiled-coil domain-containing protein 85A (CCDC85A) and complement component 8 gamma (C8G) in tumorigenesis and further investigations were required.

Further analysis of the TME in ccRCC revealed that BM-based clusters and risk groups had distinct immune landscape. Higher immune scores and infiltration levels of immune cells were discovered in the cluster or group that possessed more enriched BM-related functions. It was reported that BM was one of the barriers through which the extravasation of lymphocytes into tumor sites should be overcome (Marchand et al., 2019). During this process, laminin, the major component of BM, played an important role in mediating the functions of lymphocyte trafficking (Pozzi et al., 2017). Evidence suggested that laminins containing LAMA4 favored the transmigration of T cells through providing some permissive signals while those with LAMA5 tended to oppose this process (Sixt et al., 2001). Besides, T cells might adhere less strongly to LAMA4-bearing laminins but migrate faster across these lamins than those with LAMA5 (Zhang et al., 2020). BM distributed with a higher level of LAMA4-containing laminins would have a higher potential for lymphocyte extravasation (Wu et al., 2009). However, we discovered that cluster with high immune cell infiltration was related to poor prognosis and most of the infiltrated lymphocytes in ccRCC including CD8 T lymphocyte and CD4 T lymphocyte were associated with low survival time. This could be due to the dysfunction of lymphocytes by the suppressive lymphocytes and tumor cells. Myeloid-derived suppressor cells (MDSCs) were a cluster of immune suppressive myeloid cells frequently found in cancers (Gabrilovich et al., 2007). As their name suggested, MDSCs exerted suppressive functions on various cells, especially T lymphocytes (Gabrilovich et al., 2012). They could induce tolerance of antigen-specific T lymphocytes mainly through the production of reactive oxygen species (ROS) to nitrate receptors on T lymphocytes and reduce their responsiveness, inhibiting the anti-tumor function (Nagaraj et al., 2007). Apart from suppressive immune cells, tumor cells themselves could transduce inhibitory signals to lymphocytes for immune evasion (Muenst et al., 2016). Inhibitory immune checkpoints such as PD1 were targeted by tumor cells to transform T lymphocytes into suppressive status (Daassi et al., 2020). Thus, BM remodeling may assist in the development of the rich but suppressive immune microenvironment in ccRCC.

With an increasing understanding of the mechanisms underlying tumorigenesis, therapeutic strategies developed against them were shown to be effective in cancer therapy. ICIs, functioning through interrupting the suppressive signals transduced by CTLA4 or PD1 to reactivate the anti-tumor immunity and prevent tumor immune evasion, was approved to be successful in the treatment of multiple cancers (Sharma and Allison, 2015). It was believed that normalization of the suppressive environment and restoration of the anti-tumor immunity would be more effective than directly enhancing the immune function (Sanmamed and Chen, 2018). In line with this statement, our finding suggested that clusters with high immunogenicity potential but suppressed responded better to ICIs. The higher level of SNV, HRD, and CTA in the high risk group indicated it possessed a higher potential to generate tumor-associated neoantigens, which were critical for the immune system to exert anti-tumor function (Meng et al., 2021; van Wilpe et al., 2021; Wang et al., 2021). Meanwhile, once the suppressive environment was removed, the relatively high infiltration level of immune cells served as an immune reservoir that supported a powerful immune reaction (Waldman et al., 2020). Additionally, the increased expression of immune checkpoints in the high risk group was thought to be predictive for immunotherapy (Thommen et al., 2018). Apart from ICI monotherapy, recent studies had domonstrated the benefits of treatment combined ICI and TKI, which could have the better therapeutic capacity (Quhal et al., 2021). Our results also demonstrated that the high risk group was more sensitive to TKIs including sorafenib and sunitinib, as well as chemotherapy like mitomycin C. The mutation analysis helped discover a co-occurance mutant pair, SETD2 and PBRM1, in which SETD2 mutation was correlated with a favorable outcome of ICI-treated patients (Lu et al., 2021) and PBRM1 mutation was associated with high angiogenesis and TKIs therapeutic outcomes (Motzer et al., 2020b), indicating the potential of the high risk group for combination therapy. It was demonstrated that TKIs therapy was associated with improved vessel extravasation and enhanced drug delivery to tumors (Zhou et al., 2008; Zhou and Gallo, 2009). Moreover, after TKI treatment, the amount of suppressive immune cells including Treg and MDSCs was found to be decreased in TME (Finke et al., 2008; Ko et al., 2009). Therefore, BMRS constructed in the current study could not only help individualized administration of immunotherapy but also assist in the combination therapy of ICIs and TKIs.

In addition to the existing therapeutic agents, the investigation was also focus on novel therapeutic targets. In patients with high BMRS, we identified the upregulation of chemokine (C-X-C motif) ligand 13 (CXCL13) and its receptor CXCR5. Their interaction could impede the tumor-specific cytotoxic function of CD8 T lymphocytes and be related to the recruitment of suppressive immune cells including MDSCs and Treg (Ammirante et al., 2014). Inhibitor targeting the CXCL13/CXCR5 axis was demonstrated to have an encouraging effect on cancer treatment (Hussain et al., 2019). A Rho associated kinase inhibitor, BRD-K23875128, may be a potential therapeutic agent for high risk patients. Rho kinase pathway took part in multiple cell functions and was implicated in tumor metastasis as well as ECM remodeling. It may be a candidate for combination treatment due to its ability to increase sensitivity to other therapeutic drugs (Kim et al., 2021). As for patients with low BMRS, there may also be some curative agents. Histocompatibility leucocyte antigen E (HLA-E) and its cognate inhibitory receptor NKG2A could serve as a novel immune checkpoint to be targeted for inducing anti-tumor immunity (Borst et al., 2020). As predicted by CMap, arginase inhibitor, could also potentially treat patients with high BMRS by promoting the T cells activation and proliferation to exert an anti-tumor immune response (Borek et al., 2020).

Overall, the current study identified BM-related subtypes of ccRCC and constructed a risk scoring system BMRS for prognosis and therapeutic prediction based on both public and clinical data. The mutation, TME and treatment analysis also provided potential novel therapeutic agents for further investigations. Comparing with the existing similar gene signatures, BMRS possessed higher net benefit in predicting the prognosis of ccRCC patients. Besides, we had validated the scoring system and its gene expression using external dataset and clinical samples. However, the lack of accessibility to clinical treatment-related data restricted our analysis of BMRS in predicting therapeutic response. More in-depth study could be conducted to validate the proposed potential therapeutic targets for ccRCC.

## 5 Conclusion

In summary, we recognized BM-related subtypes of ccRCC with distinct survival and TME features. A risk scoring system BMRS was established for prognosis prediction and individualized treatment instruction. Mechanistic investigations based on BM-related clusters and risk groups helped identified some therapeutic candidates. With more and more studies focusing on combination therapy, our results may provide certain practical instructions for clinical application and future research.

## Data Availability

The data and information demonstrated and analyzed throughout the present study were obtained from the Genomics Data Commons Data Portal (https://portal.gdc.cancer.gov/), ArrayExpress (https://www.ebi.ac.uk/arrayexpress/), Gene Set Enrichment Analysis (http://www.gsea-msigdb.org/gsea/index.jsp), xCell (https://xcell.ucsf.edu/), The Cancer Immunome Database (https://tcia.at/home), Genomics of Drug Sensitivity in Cancer (https://www.cancerrxgene.org), ConnectivityMap (CMap, https://clue.io). The patient information of the clinical transcriptome data could be accessed from the supplementary material (Supplementary Table S1).

## Abbreviation

ccRCC, Clear cell renal cell carcinoma; ICIs, Immune checkpoint inhibitors; VEGF, Vascular endothelial growth factor; TKIs, Tyrosine kinase inhibitors; TME, Tumor microenvironment; BM, Basement membrane; ECM, Extracellular matrix; TCGA, The cancer genome atlas; TPM, Transcripts per million; CNV, Copy number variations; LASSO, Least absolute selection and shrinkage operator; BMRS, BM-related risk score system; CTA, Cancer testis antigens; HRD, Homologous recombination repair deficiency; ITH, Intratumoral heterogeneity; SNV, Single-nucleotide variation; TIDE, Tumor immune dysfunction and evasion; TMB, Tumor mutation burden; MDSC, Myeloid-derived suppressor cell; Treg, Regulatory T cell; WGCNA, Weight gene correlation network analysis; CNMF, Non-negative matrix factorization; ESTIMATE, Estimation of stromal and immune cells in malignant tumor tissues using expression data; ssGSEA, Single-sample gene set enrichment analysis.
