# Supplementary material for: Identification of a basement membrane-based risk scoring system for prognosis prediction and individualized therapy in clear cell renal cell carcinoma
Source: Front Genet. 2023 Feb 3;14:1038924. doi: 10.3389/fgene.2023.1038924 (PMC9935575; doi:10.3389/fgene.2023.1038924)
Supplement: Supplementary file 1 [file Table1.DOCX]

| Table S1. Genes differentially expressed in ccRCC. | | |
| --- | --- | --- |
| Gene | logFC | pValue |
| COL4A3 | -1.83246 | 3.14E-34 |
| ITGAX | 3.235601 | 9.29E-38 |
| ITGAM | 2.076552 | 1.67E-29 |
| HMCN1 | 1.399847 | 3.30E-14 |
| MMP21 | 1.028577 | 0.000157 |
| CD44 | 1.393811 | 8.76E-19 |
| ANG | 1.778622 | 4.04E-26 |
| FREM3 | -1.31181 | 2.26E-20 |
| FN1 | 1.822088 | 4.80E-20 |
| EGFLAM | 1.618397 | 1.04E-16 |
| THBS2 | 1.441481 | 3.19E-07 |
| LOXL2 | 2.619085 | 4.86E-31 |
| LUM | -1.20793 | 1.30E-16 |
| ADAMTS4 | 2.017817 | 1.36E-22 |
| COL8A1 | 2.08082 | 1.55E-23 |
| NID2 | 1.156987 | 1.02E-08 |
| SLIT2 | -1.10995 | 2.09E-13 |
| LAMC2 | -1.56372 | 1.13E-19 |
| FREM1 | -3.31866 | 2.37E-38 |
| SLIT1 | 1.150079 | 7.36E-15 |
| FGF9 | -3.25228 | 8.20E-37 |
| ADAMTS14 | 3.32416 | 2.47E-30 |
| MATN2 | -1.00769 | 2.22E-19 |
| ITGA5 | 1.976736 | 2.14E-29 |
| MEP1B | 1.8565 | 5.54E-09 |
| P3H1 | 1.36245 | 8.05E-31 |
| FREM2 | -1.4509 | 2.16E-29 |
| COL6A2 | 2.008519 | 7.76E-27 |
| OGN | -1.00744 | 6.19E-15 |
| NELL2 | 1.507762 | 1.44E-10 |
| ITGA2B | 1.424723 | 0.019748 |
| ADAMTS15 | -1.98285 | 2.09E-21 |
| MATN1 | 1.682126 | 9.21E-23 |
| VWA1 | 1.392503 | 8.28E-20 |
| HAPLN1 | 3.406576 | 8.53E-30 |
| LAMA2 | -1.34658 | 2.27E-19 |
| SPARCL1 | 1.687932 | 8.12E-20 |
| ADAMTS8 | -1.17566 | 9.79E-28 |
| COL4A1 | 1.947092 | 1.41E-26 |
| FBLN5 | -1.60033 | 5.45E-29 |
| TGFBI | 2.702486 | 2.01E-23 |
| BCAN | 1.959011 | 1.02E-26 |
| EFEMP2 | 1.008669 | 8.96E-20 |
| EVA1C | 1.155727 | 8.23E-22 |
| MMP17 | 2.057414 | 1.06E-08 |
| POSTN | 1.765366 | 1.35E-13 |
| TIMP1 | 1.95819 | 9.62E-26 |
| ADAMTS16 | -2.42892 | 7.80E-30 |
| GPC3 | -3.83806 | 1.45E-40 |
| VCAN | 1.855244 | 2.23E-17 |
| NELL1 | -5.73225 | 5.36E-42 |
| ITGB6 | -2.61997 | 7.73E-30 |
| MEP1A | 2.984364 | 2.08E-25 |
| DCN | -2.42518 | 9.48E-33 |
| SPARC | 1.984214 | 1.52E-30 |
| COL4A4 | -2.03715 | 9.73E-38 |
| TENM1 | 2.648001 | 1.24E-26 |
| HSPG2 | 1.039374 | 1.76E-06 |
| MMP7 | -1.07588 | 4.26E-10 |
| SERPINF1 | 1.088324 | 5.86E-07 |
| NID1 | 1.495984 | 7.28E-21 |
| COL6A3 | 1.404446 | 6.97E-11 |
| BGN | 1.101177 | 3.29E-11 |
| GPC2 | 2.02907 | 8.92E-25 |
| COL9A3 | -1.38866 | 1.42E-19 |
| ADAMTS2 | 1.950989 | 8.35E-25 |
| FBLN1 | -1.5519 | 9.41E-23 |
| LAMB4 | -1.44458 | 2.53E-33 |
| ADAMTS20 | 5.631656 | 2.01E-26 |
| COL9A2 | -2.12837 | 1.38E-32 |
| ITGA4 | 1.910238 | 4.35E-23 |
| ITGA10 | 1.252758 | 2.43E-08 |
| NTN4 | -1.59208 | 5.85E-26 |
| THBS4 | 2.388006 | 3.27E-05 |
| ADAMTS7 | 2.592351 | 8.72E-35 |
| VWA2 | -2.90467 | 9.37E-34 |
| FRAS1 | -1.0714 | 2.26E-22 |
| MMP14 | 1.514508 | 5.72E-26 |
| DAG1 | -1.03993 | 2.65E-31 |
| SEMA3B | -1.27939 | 6.72E-28 |
| COL15A1 | 1.53539 | 9.09E-17 |
| ADAMTS10 | 2.233398 | 2.82E-27 |
| LAD1 | -2.56448 | 1.70E-36 |
| ACHE | 1.213662 | 0.000556 |
| COL4A2 | 1.611619 | 6.51E-24 |
| COL4A5 | -2.17607 | 2.69E-37 |
| ADAMTS19 | -4.11548 | 1.49E-24 |
| TNC | -1.1048 | 2.22E-05 |
| ITGA7 | 1.062301 | 3.91E-16 |
| NPNT | -1.80908 | 2.40E-29 |
| UNC5B | 1.946657 | 7.45E-26 |
| LAMA4 | 2.725209 | 5.09E-35 |
| COL14A1 | -1.18624 | 1.10E-11 |
| GPC5 | -5.70771 | 5.94E-43 |
| ITGB2 | 2.446005 | 4.20E-33 |
| LOXL4 | -1.17734 | 4.59E-08 |
| ADAMTS18 | 1.473351 | 5.01E-13 |
| PXDN | 1.748873 | 2.24E-23 |
| COL6A1 | 1.218791 | 8.24E-18 |
| COL4A6 | -4.07019 | 2.18E-40 |
| CSPG4 | 2.888684 | 9.81E-33 |
| COL5A1 | 2.059194 | 5.30E-18 |
| TLL1 | 1.721643 | 6.21E-14 |
| UNC5A | 3.387423 | 2.74E-28 |
| SPON2 | 1.676303 | 5.34E-18 |
| ACAN | 2.664056 | 1.97E-24 |
