# Supplementary figures and images for: Identification of a basement membrane-based risk scoring system for prognosis prediction and individualized therapy in clear cell renal cell carcinoma
Source: Front Genet. 2023 Feb 3;14:1038924. doi: 10.3389/fgene.2023.1038924 (PMC9935575; doi:10.3389/fgene.2023.1038924)

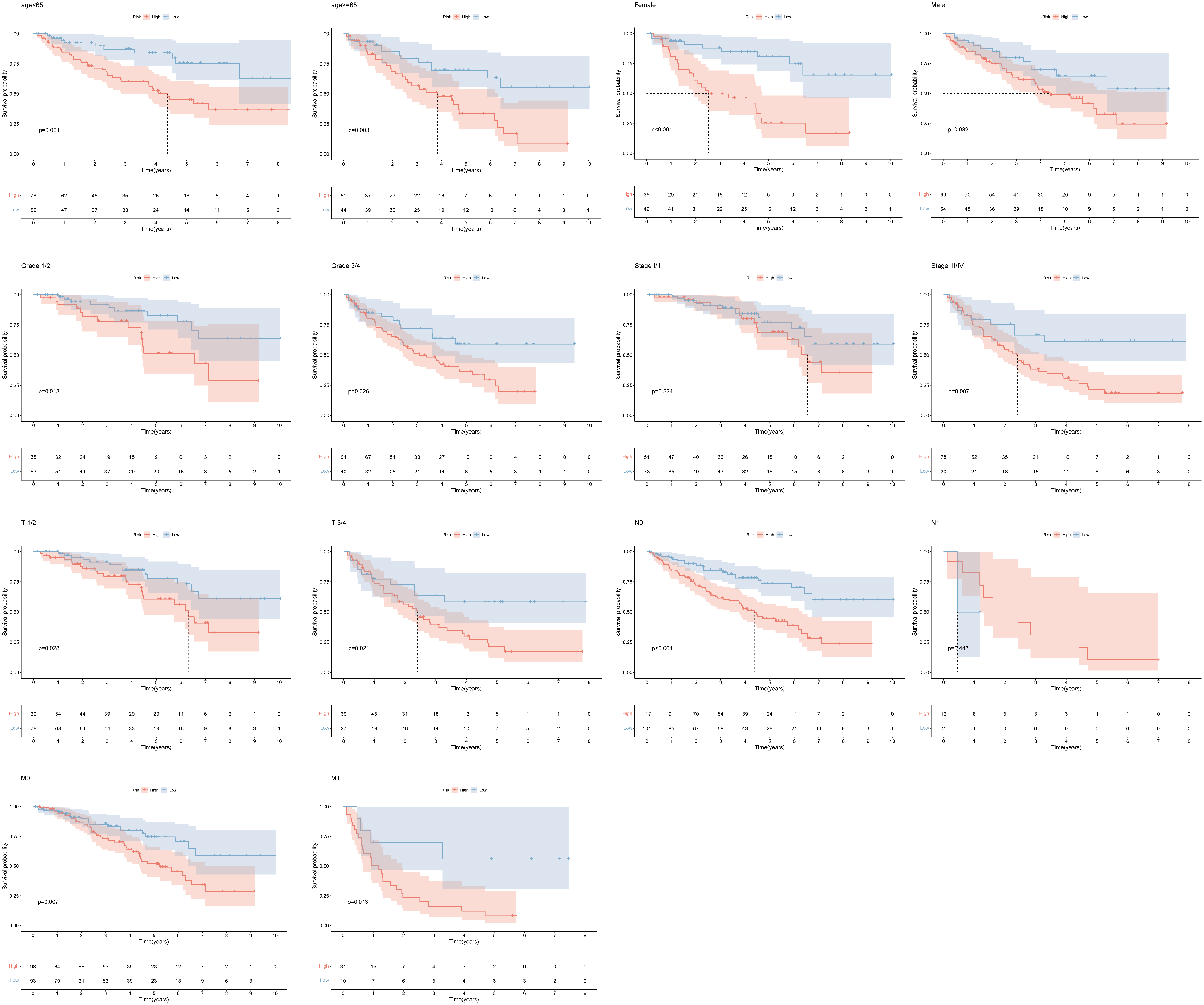

Supplement: Supplementary file 2 [file Image3.TIF]

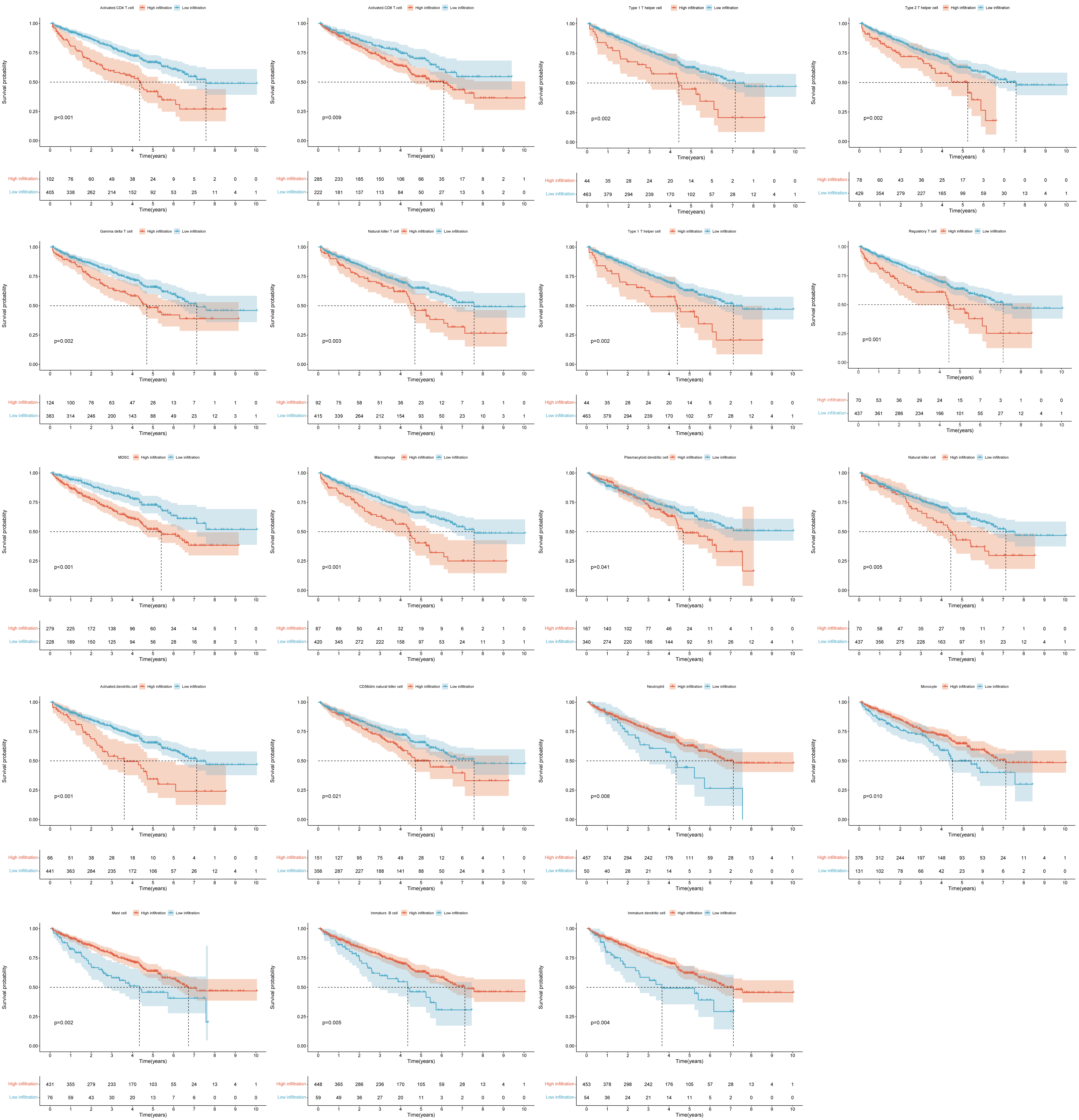

Supplement: Supplementary file 3 [file Image4.TIF]

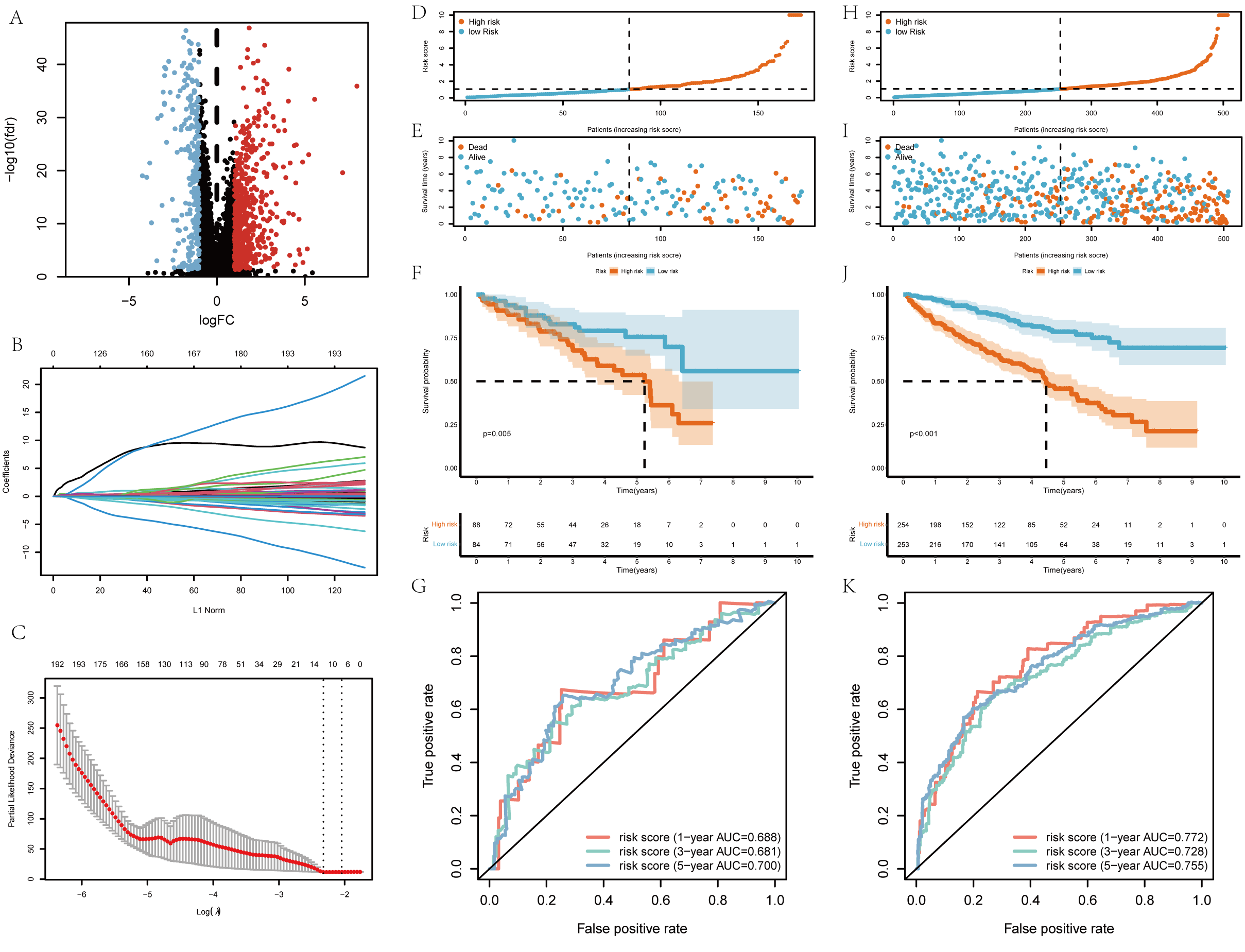

Supplement: Supplementary file 5 [file Image2.TIF]

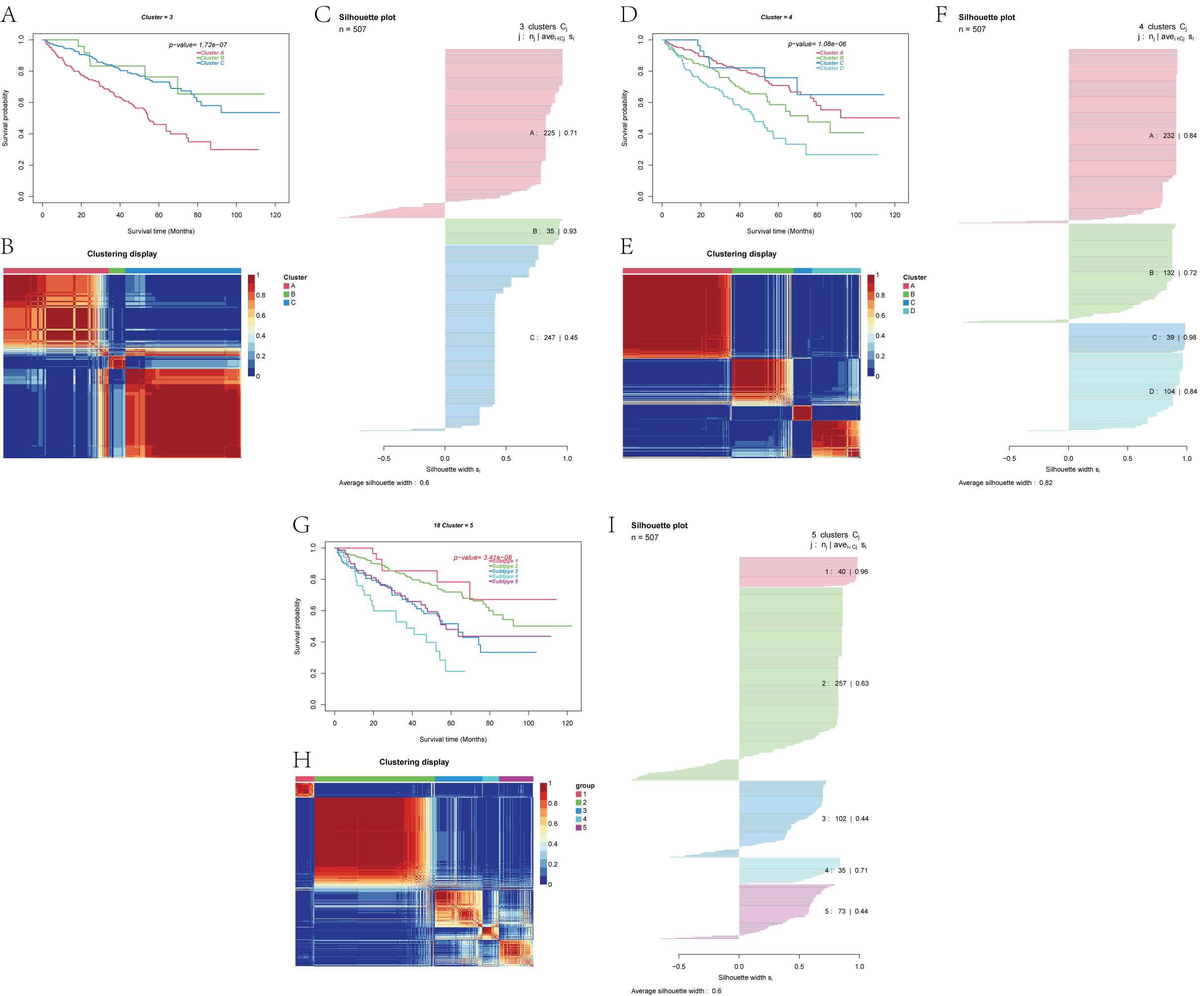

Supplement: Supplementary file 6 [file Image1.TIF]
