# Supplementary material for: Identification of a basement membrane-based risk scoring system for prognosis prediction and individualized therapy in clear cell renal cell carcinoma
Source: Front Genet. 2023 Feb 3;14:1038924. doi: 10.3389/fgene.2023.1038924 (PMC9935575; doi:10.3389/fgene.2023.1038924)
Supplement: Supplementary file 7 [file Table2.DOCX]

| Table S2. Results of univariate cox regression analysis of the differentially expressed BM genes. | | | | |
| --- | --- | --- | --- | --- |
| Gene | HR | HR.95L | HR.95H | pvalue |
| COL4A3 | 0.763946 | 0.659432 | 0.885025 | 0.000334 |
| ITGAX | 1.245261 | 1.058074 | 1.465563 | 0.008311 |
| HMCN1 | 0.731057 | 0.627422 | 0.851811 | 5.91E-05 |
| CD44 | 1.353108 | 1.158612 | 1.580254 | 0.000134 |
| ANG | 0.863516 | 0.771762 | 0.966178 | 0.010459 |
| LUM | 1.079135 | 1.009836 | 1.153189 | 0.024513 |
| FREM1 | 0.562546 | 0.44241 | 0.715304 | 2.69E-06 |
| ADAMTS14 | 1.696817 | 1.473515 | 1.953958 | 2.06E-13 |
| MATN2 | 0.832962 | 0.727071 | 0.954275 | 0.008423 |
| P3H1 | 1.747307 | 1.413519 | 2.159915 | 2.47E-07 |
| FREM2 | 0.694065 | 0.61077 | 0.788719 | 2.16E-08 |
| COL6A2 | 1.256606 | 1.087416 | 1.452119 | 0.001963 |
| ITGA2B | 1.199479 | 1.027643 | 1.400047 | 0.021132 |
| MATN1 | 4.009847 | 2.057199 | 7.815907 | 4.54E-05 |
| VWA1 | 0.773195 | 0.681024 | 0.877841 | 7.13E-05 |
| SPARCL1 | 0.792631 | 0.711631 | 0.882852 | 2.39E-05 |
| COL4A1 | 0.848664 | 0.748081 | 0.962772 | 0.010791 |
| TGFBI | 1.113674 | 1.040226 | 1.192307 | 0.001982 |
| MMP17 | 1.51023 | 1.286213 | 1.773264 | 4.84E-07 |
| TIMP1 | 1.475042 | 1.269255 | 1.714193 | 3.98E-07 |
| ADAMTS16 | 0.806331 | 0.666624 | 0.975317 | 0.026594 |
| MEP1A | 0.408478 | 0.180372 | 0.925055 | 0.031813 |
| SPARC | 0.819204 | 0.704104 | 0.95312 | 0.009837 |
| COL4A4 | 0.628664 | 0.539733 | 0.732249 | 2.45E-09 |
| HSPG2 | 0.792852 | 0.720875 | 0.872015 | 1.75E-06 |
| MMP7 | 1.077055 | 1.010954 | 1.147477 | 0.021613 |
| SERPINF1 | 1.304553 | 1.172599 | 1.451355 | 1.03E-06 |
| NID1 | 0.873345 | 0.766951 | 0.994498 | 0.041032 |
| COL6A3 | 1.176707 | 1.056872 | 1.310129 | 0.002984 |
| GPC2 | 1.911472 | 1.400506 | 2.608861 | 4.46E-05 |
| FBLN1 | 1.150065 | 1.033033 | 1.280355 | 0.010665 |
| COL9A2 | 1.24951 | 1.087776 | 1.435291 | 0.001635 |
| ITGA4 | 0.84722 | 0.741605 | 0.967875 | 0.014662 |
| NTN4 | 0.731619 | 0.658194 | 0.813235 | 6.99E-09 |
| FRAS1 | 0.76174 | 0.672179 | 0.863234 | 2.00E-05 |
| COL15A1 | 0.815404 | 0.726969 | 0.914597 | 0.000494 |
| ADAMTS10 | 1.294101 | 1.108877 | 1.510265 | 0.001071 |
| LAD1 | 0.904434 | 0.821951 | 0.995194 | 0.039522 |
| ACHE | 1.342602 | 1.181185 | 1.526077 | 6.55E-06 |
| COL4A2 | 0.868947 | 0.758313 | 0.995721 | 0.043209 |
| COL4A5 | 1.24 | 1.059215 | 1.451642 | 0.007462 |
| ITGA7 | 0.844961 | 0.719825 | 0.99185 | 0.039396 |
| NPNT | 0.698995 | 0.619305 | 0.788938 | 6.69E-09 |
| UNC5B | 0.835025 | 0.735501 | 0.948016 | 0.005362 |
| COL6A1 | 1.370614 | 1.162334 | 1.616217 | 0.000178 |
| CSPG4 | 0.876289 | 0.779925 | 0.984559 | 0.026298 |
| COL5A1 | 1.220489 | 1.093288 | 1.362491 | 0.000388 |
| TLL1 | 0.731481 | 0.643127 | 0.831973 | 1.93E-06 |
| SPON2 | 1.19549 | 1.07062 | 1.334923 | 0.001512 |
