# Supplementary material for: Identification of a basement membrane-based risk scoring system for prognosis prediction and individualized therapy in clear cell renal cell carcinoma
Source: Front Genet. 2023 Feb 3;14:1038924. doi: 10.3389/fgene.2023.1038924 (PMC9935575; doi:10.3389/fgene.2023.1038924)
Supplement: Supplementary file 8 [file Table3.DOCX]

| Table S3. Results of the univariate cox regression analysis of differentially expressed genes between BM clusters. | | | | |
| --- | --- | --- | --- | --- |
| Gene | HR | HR.95L | HR.95H | pvalue |
| CPN2 | 1.0041 | 1.001458 | 1.00675 | 0.002339 |
| RDH16 | 1.565487 | 1.268099 | 1.932618 | 3.05E-05 |
| NKX3-2 | 1.286504 | 1.161848 | 1.424535 | 1.27E-06 |
| GPR84 | 1.250062 | 1.162242 | 1.344517 | 1.91E-09 |
| KCNV1 | 0.768503 | 0.605615 | 0.975201 | 0.030266 |
| MSRA | 0.98027 | 0.967634 | 0.993071 | 0.00261 |
| NTNG2 | 1.186933 | 1.093654 | 1.288167 | 4.06E-05 |
| NEK2 | 1.090327 | 1.05602 | 1.125748 | 1.15E-07 |
| PODNL1 | 1.043407 | 1.016603 | 1.070918 | 0.001374 |
| CDCA3 | 1.093357 | 1.062477 | 1.125134 | 1.02E-09 |
| GPR45 | 1.59868 | 1.244806 | 2.053154 | 0.000238 |
| CDS1 | 0.960006 | 0.939714 | 0.980735 | 0.000181 |
| AIF1L | 0.995711 | 0.992517 | 0.998915 | 0.008739 |
| MPP2 | 1.180573 | 1.072511 | 1.299524 | 0.000701 |
| WNT7B | 1.056579 | 1.03242 | 1.081303 | 3.11E-06 |
| SLC6A19 | 0.990628 | 0.98468 | 0.996612 | 0.00218 |
| STEAP3 | 1.004187 | 1.00163 | 1.006751 | 0.00132 |
| PDIA2 | 1.140635 | 1.08789 | 1.195936 | 5.11E-08 |
| ZIC2 | 1.223757 | 1.129644 | 1.325711 | 7.59E-07 |
| CDCA8 | 1.026742 | 1.015196 | 1.038419 | 4.79E-06 |
| PTPRH | 1.023292 | 1.007651 | 1.039176 | 0.003392 |
| PITX1 | 1.034929 | 1.01139 | 1.059015 | 0.003446 |
| KIF2C | 1.048694 | 1.031301 | 1.06638 | 2.52E-08 |
| XIRP1 | 1.087992 | 1.032062 | 1.146953 | 0.001736 |
| GRIN2D | 1.065314 | 1.020713 | 1.111864 | 0.003738 |
| LAIR2 | 1.0132 | 1.002437 | 1.024079 | 0.016097 |
| CPNE7 | 1.02647 | 1.008666 | 1.044587 | 0.003427 |
| ABCG2 | 0.952166 | 0.930945 | 0.97387 | 2.02E-05 |
| IL20RB | 1.002379 | 1.000612 | 1.004149 | 0.008317 |
| MYL3 | 0.956697 | 0.936124 | 0.977723 | 6.58E-05 |
| IL11 | 1.065976 | 1.037912 | 1.094798 | 2.68E-06 |
| C6orf223 | 0.993049 | 0.987762 | 0.998364 | 0.010434 |
| CLVS2 | 0.329062 | 0.125557 | 0.862414 | 0.023755 |
| CRP | 1.000921 | 1.000242 | 1.0016 | 0.007828 |
| KIF18B | 1.086842 | 1.056286 | 1.118282 | 1.04E-08 |
| E2F7 | 1.138347 | 1.082665 | 1.196891 | 4.10E-07 |
| JSRP1 | 1.035798 | 1.010401 | 1.061834 | 0.005488 |
| CARD14 | 1.118992 | 1.055368 | 1.186452 | 0.000167 |
| CD44 | 1.002836 | 1.001528 | 1.004145 | 2.10E-05 |
| KCNN4 | 1.130306 | 1.079139 | 1.1839 | 2.19E-07 |
| SCN4A | 0.880143 | 0.809828 | 0.956563 | 0.002653 |
| PLAUR | 1.015928 | 1.010712 | 1.021171 | 1.78E-09 |
| CHST6 | 1.300368 | 1.124415 | 1.503855 | 0.000399 |
| UBE2C | 1.00722 | 1.004505 | 1.009943 | 1.76E-07 |
| OR10Q1 | 0.438399 | 0.225995 | 0.850432 | 0.014721 |
| TM4SF19 | 1.049057 | 1.02296 | 1.07582 | 0.000194 |
| FN1 | 1.000468 | 1.000074 | 1.000863 | 0.019968 |
| SLC5A12 | 0.990261 | 0.985033 | 0.995518 | 0.000291 |
| TMEM72 | 0.989722 | 0.98049 | 0.999041 | 0.030728 |
| CCDC85A | 0.711629 | 0.62165 | 0.814631 | 8.12E-07 |
| RNASE2 | 1.062768 | 1.038183 | 1.087934 | 3.43E-07 |
| PYCR1 | 1.011861 | 1.006804 | 1.016944 | 3.98E-06 |
| SERPINE1 | 1.000265 | 1.000131 | 1.000398 | 0.000104 |
| CST1 | 1.055827 | 1.005017 | 1.109205 | 0.030861 |
| ADGRV1 | 0.08944 | 0.022122 | 0.361608 | 0.000706 |
| ARL4C | 1.002253 | 1.000519 | 1.003991 | 0.010874 |
| APBA2 | 1.085403 | 1.023286 | 1.151289 | 0.006419 |
| GTSF1 | 1.134943 | 1.039287 | 1.239403 | 0.004836 |
| ETV4 | 1.041901 | 1.008891 | 1.07599 | 0.012459 |
| ZIC5 | 1.572561 | 1.262118 | 1.959363 | 5.47E-05 |
| PDGFRA | 1.015947 | 1.005902 | 1.026092 | 0.001804 |
| THBS2 | 1.001931 | 1.000421 | 1.003444 | 0.012193 |
| MMP9 | 1.001047 | 1.000348 | 1.001746 | 0.003302 |
| OCLN | 0.896004 | 0.837444 | 0.958659 | 0.001451 |
| PYGO1 | 1.150515 | 1.061436 | 1.24707 | 0.000649 |
| ESRRG | 0.860309 | 0.796089 | 0.92971 | 0.000144 |
| EPHX2 | 0.987918 | 0.980447 | 0.995446 | 0.001698 |
| LUM | 1.000811 | 1.000402 | 1.00122 | 0.000101 |
| NUF2 | 1.096682 | 1.060587 | 1.134006 | 6.49E-08 |
| SYN1 | 1.063942 | 1.02452 | 1.104882 | 0.001293 |
| SH3GL3 | 1.498355 | 1.117833 | 2.008412 | 0.006827 |
| ZP1 | 1.053006 | 1.013696 | 1.09384 | 0.007797 |
| PRAME | 1.00526 | 1.001929 | 1.008602 | 0.001948 |
| ERC2 | 1.896468 | 1.248748 | 2.880158 | 0.002683 |
| RGCC | 0.996813 | 0.995445 | 0.998183 | 5.27E-06 |
| HJURP | 1.07335 | 1.050945 | 1.096232 | 4.80E-11 |
| TOX3 | 0.935704 | 0.893746 | 0.979631 | 0.004523 |
| MAGEA10 | 1.180131 | 1.020446 | 1.364804 | 0.025561 |
| TRNP1 | 1.011916 | 1.001331 | 1.022613 | 0.027255 |
| LDHD | 0.982556 | 0.96687 | 0.998496 | 0.032091 |
| C6orf118 | 2.399902 | 1.397665 | 4.120821 | 0.001505 |
| PANK1 | 0.902705 | 0.868124 | 0.938665 | 2.81E-07 |
| COL1A1 | 1.000114 | 1.00004 | 1.000189 | 0.002632 |
| PADI3 | 1.009948 | 1.001046 | 1.018929 | 0.028416 |
| KIF4A | 1.039571 | 1.022842 | 1.056573 | 2.75E-06 |
| P4HA3 | 1.024717 | 1.01665 | 1.032848 | 1.41E-09 |
| TNFSF14 | 1.035792 | 1.018979 | 1.052882 | 2.54E-05 |
| RERGL | 0.96524 | 0.934512 | 0.996978 | 0.032088 |
| LRRC19 | 0.96428 | 0.947801 | 0.981046 | 3.54E-05 |
| PCCA | 0.952458 | 0.932693 | 0.972642 | 5.30E-06 |
| AURKB | 1.034974 | 1.02239 | 1.047712 | 3.63E-08 |
| TAC3 | 1.643354 | 1.133054 | 2.38348 | 0.008834 |
| C11orf86 | 1.006097 | 1.000289 | 1.011938 | 0.039599 |
| CA4 | 0.969622 | 0.951203 | 0.988396 | 0.001617 |
| WT1 | 1.024112 | 1.004899 | 1.043692 | 0.013676 |
| BTBD11 | 1.061291 | 1.026243 | 1.097536 | 0.000517 |
| PDGFD | 0.989107 | 0.984679 | 0.993554 | 1.71E-06 |
| FREM1 | 0.875854 | 0.775736 | 0.988894 | 0.032332 |
| ANXA13 | 0.98449 | 0.971722 | 0.997426 | 0.018932 |
| HPX | 1.027258 | 1.000962 | 1.054245 | 0.04209 |
| FDCSP | 1.000977 | 1.000587 | 1.001367 | 8.97E-07 |
| CDH9 | 0.742101 | 0.566464 | 0.972195 | 0.030418 |
| GRIK3 | 0.964485 | 0.940086 | 0.989517 | 0.005674 |
| FAM107A | 0.963824 | 0.946529 | 0.981435 | 6.65E-05 |
| CLCN5 | 0.969265 | 0.956201 | 0.982508 | 6.52E-06 |
| NEBL | 0.961057 | 0.940046 | 0.982538 | 0.000429 |
| NFE4 | 1.008149 | 1.003675 | 1.012643 | 0.000348 |
| SEMA3A | 1.056464 | 1.024971 | 1.088924 | 0.000375 |
| ORM1 | 1.020939 | 1.012409 | 1.029542 | 1.29E-06 |
| GJB1 | 0.976342 | 0.964216 | 0.98862 | 0.000173 |
| F11 | 0.817185 | 0.707818 | 0.943452 | 0.005887 |
| FBP1 | 0.992954 | 0.989103 | 0.99682 | 0.000362 |
| LARGE2 | 0.973395 | 0.94985 | 0.997524 | 0.030896 |
| C1QL4 | 0.985502 | 0.972197 | 0.99899 | 0.035227 |
| ADAMTS14 | 1.087917 | 1.053438 | 1.123525 | 2.93E-07 |
| NPY4R | 3.325026 | 1.979054 | 5.586404 | 5.66E-06 |
| RYR2 | 1.112539 | 1.018055 | 1.215792 | 0.018516 |
| EN1 | 1.071323 | 1.022607 | 1.122361 | 0.003715 |
| SLC22A12 | 0.99632 | 0.993261 | 0.999388 | 0.018782 |
| AJAP1 | 0.745287 | 0.659497 | 0.842237 | 2.46E-06 |
| CEBPB | 1.005472 | 1.002538 | 1.008415 | 0.000253 |
| SLC47A1 | 0.994523 | 0.992201 | 0.99685 | 4.11E-06 |
| MUC12 | 1.528502 | 1.276729 | 1.829926 | 3.83E-06 |
| ACAD11 | 0.869801 | 0.818728 | 0.92406 | 6.24E-06 |
| GPAT3 | 0.983483 | 0.96758 | 0.999648 | 0.045257 |
| PABPC4L | 0.9003 | 0.856395 | 0.946455 | 3.83E-05 |
| KCNJ15 | 0.981349 | 0.974179 | 0.988572 | 4.85E-07 |
| CHI3L2 | 1.014054 | 1.003578 | 1.02464 | 0.008439 |
| PLCL1 | 0.901666 | 0.860524 | 0.944776 | 1.40E-05 |
| IL2RA | 1.032719 | 1.018955 | 1.046669 | 2.57E-06 |
| CLEC18C | 0.968172 | 0.94384 | 0.99313 | 0.012746 |
| ADAMDEC1 | 1.019234 | 1.006693 | 1.03193 | 0.002561 |
| ANLN | 1.025513 | 1.01701 | 1.034086 | 3.02E-09 |
| PTH1R | 0.983466 | 0.972228 | 0.994835 | 0.004469 |
| DIRAS2 | 0.973416 | 0.959268 | 0.987772 | 0.00031 |
| TUBB3 | 1.081032 | 1.005611 | 1.16211 | 0.034721 |
| CCIN | 1.437027 | 1.149634 | 1.796265 | 0.001448 |
| ADH1C | 1.022887 | 1.004556 | 1.041552 | 0.014182 |
| PLA2G4D | 1.181814 | 1.039409 | 1.34373 | 0.010773 |
| GJB2 | 1.004539 | 1.001728 | 1.007357 | 0.001539 |
| CDH15 | 1.153662 | 1.050137 | 1.267394 | 0.002885 |
| HAMP | 1.082127 | 1.048594 | 1.116732 | 8.91E-07 |
| FREM2 | 0.895756 | 0.849912 | 0.944073 | 4.01E-05 |
| CLIC3 | 1.03979 | 1.005603 | 1.075139 | 0.022164 |
| TRIB3 | 1.004634 | 1.002282 | 1.006991 | 0.000111 |
| BIRC5 | 1.028221 | 1.017185 | 1.039376 | 4.31E-07 |
| COL6A2 | 1.000567 | 1.000176 | 1.000958 | 0.004506 |
| F2RL3 | 0.97958 | 0.969576 | 0.989688 | 8.18E-05 |
| CYP26A1 | 1.329227 | 1.101592 | 1.603902 | 0.002983 |
| SLC17A4 | 0.994213 | 0.988595 | 0.999863 | 0.04473 |
| PDGFRL | 1.009139 | 1.002431 | 1.015893 | 0.007509 |
| TMEM132A | 1.023422 | 1.012111 | 1.03486 | 4.45E-05 |
| TEX19 | 1.185713 | 1.036333 | 1.356625 | 0.013159 |
| AVPR1B | 0.933299 | 0.895264 | 0.97295 | 0.001147 |
| CYP2J2 | 0.998169 | 0.996709 | 0.999631 | 0.014099 |
| KERA | 1.034207 | 1.008846 | 1.060206 | 0.007925 |
| APOLD1 | 0.993644 | 0.990939 | 0.996357 | 4.57E-06 |
| KRT78 | 2.158128 | 1.139611 | 4.086937 | 0.018221 |
| ADGRG3 | 1.063343 | 1.024145 | 1.104041 | 0.001351 |
| TRPV3 | 1.434597 | 1.174258 | 1.752654 | 0.000412 |
| WNT10B | 1.44443 | 1.182926 | 1.763743 | 0.000308 |
| PGLYRP4 | 42.01323 | 4.567546 | 386.4465 | 0.000961 |
| C1S | 1.001654 | 1.00084 | 1.002469 | 6.73E-05 |
| SCGB1D2 | 0.958614 | 0.927029 | 0.991275 | 0.013413 |
| MAPT | 0.971932 | 0.957475 | 0.986608 | 0.000197 |
| CBS | 2.258135 | 1.494767 | 3.411351 | 0.000109 |
| IYD | 0.48408 | 0.329685 | 0.710781 | 0.000214 |
| GAS1 | 1.006781 | 1.002199 | 1.011385 | 0.003686 |
| ORM2 | 1.035338 | 1.017725 | 1.053256 | 7.28E-05 |
| BARX1 | 1.026772 | 1.012331 | 1.041419 | 0.000256 |
| F2 | 1.007273 | 1.001291 | 1.013291 | 0.017108 |
| CXCL5 | 1.004918 | 1.002624 | 1.007217 | 2.58E-05 |
| ENAM | 0.928312 | 0.869727 | 0.990843 | 0.025315 |
| ASGR1 | 1.094629 | 1.028265 | 1.165276 | 0.004605 |
| KIF14 | 1.241563 | 1.153521 | 1.336326 | 8.13E-09 |
| C10orf55 | 1.422718 | 1.161302 | 1.742981 | 0.000665 |
| FSTL4 | 0.697935 | 0.494148 | 0.985764 | 0.041216 |
| PLAC1 | 1.551018 | 1.251366 | 1.922426 | 6.14E-05 |
| KCNH3 | 1.09579 | 1.011763 | 1.186795 | 0.024624 |
| PLAU | 1.002951 | 1.001211 | 1.004694 | 0.000879 |
| TNFAIP6 | 0.997613 | 0.995449 | 0.999781 | 0.030938 |
| CLEC18B | 0.985992 | 0.976825 | 0.995245 | 0.003074 |
| FUT6 | 0.963417 | 0.945784 | 0.981379 | 7.67E-05 |
| CYP4A11 | 0.997155 | 0.994838 | 0.999477 | 0.016345 |
| RGS17 | 1.314578 | 1.176259 | 1.469161 | 1.42E-06 |
| SLC22A4 | 0.968878 | 0.951117 | 0.98697 | 0.00081 |
| VGLL3 | 1.045133 | 1.00591 | 1.085886 | 0.023705 |
| CLIC5 | 0.918974 | 0.875202 | 0.964934 | 0.00069 |
| BSPRY | 0.943153 | 0.913552 | 0.973713 | 0.000322 |
| CDKL2 | 0.847334 | 0.786969 | 0.91233 | 1.12E-05 |
| CPA4 | 1.013324 | 1.00636 | 1.020336 | 0.000169 |
| UCHL1 | 1.003996 | 1.001448 | 1.00655 | 0.002096 |
| COL11A1 | 1.004447 | 1.00168 | 1.007222 | 0.001616 |
| SULT1C4 | 0.990003 | 0.983516 | 0.996533 | 0.002741 |
| TNN | 0.805568 | 0.674371 | 0.962288 | 0.017135 |
| LOX | 1.000444 | 1.000046 | 1.000842 | 0.028904 |
| IL31RA | 1.156362 | 1.080571 | 1.237468 | 2.66E-05 |
| MTTP | 1.026831 | 1.012653 | 1.041208 | 0.00019 |
| HAS2 | 1.066175 | 1.037403 | 1.095745 | 4.42E-06 |
| TPX2 | 1.0115 | 1.007107 | 1.015912 | 2.61E-07 |
| CD7 | 1.03545 | 1.01365 | 1.057719 | 0.001333 |
| RRM2 | 1.032139 | 1.020822 | 1.043582 | 1.87E-08 |
| CILP2 | 1.008328 | 1.001267 | 1.01544 | 0.020722 |
| CLMP | 1.02038 | 1.012551 | 1.02827 | 2.84E-07 |
| SPOCD1 | 1.191416 | 1.07931 | 1.315167 | 0.000513 |
| FBXL16 | 0.985658 | 0.97803 | 0.993346 | 0.000268 |
| STYK1 | 1.223246 | 1.027564 | 1.456192 | 0.02347 |
| COL1A2 | 1.000355 | 1.000139 | 1.00057 | 0.001273 |
| MELTF | 1.00936 | 1.003311 | 1.015446 | 0.002385 |
| SLC5A8 | 0.989483 | 0.983082 | 0.995927 | 0.001411 |
| SOWAHB | 0.905865 | 0.87232 | 0.940699 | 2.82E-07 |
| CASR | 0.67287 | 0.516322 | 0.876883 | 0.003364 |
| AADAC | 1.09905 | 1.030186 | 1.172518 | 0.004226 |
| CCL11 | 1.038663 | 1.01609 | 1.061737 | 0.000715 |
| LPA | 0.150641 | 0.030692 | 0.739354 | 0.019701 |
| EDNRB | 0.993423 | 0.990672 | 0.996182 | 3.12E-06 |
| PLK1 | 1.063081 | 1.041031 | 1.085599 | 1.06E-08 |
| KL | 0.988484 | 0.983155 | 0.993842 | 2.67E-05 |
| CORO6 | 1.096944 | 1.043231 | 1.153422 | 0.000304 |
| CCNO | 1.077813 | 1.016383 | 1.142956 | 0.012325 |
| GBX2 | 1.680562 | 1.186871 | 2.379609 | 0.00344 |
| ADAM12 | 1.022228 | 1.01163 | 1.032937 | 3.56E-05 |
| PPP1R1A | 1.002302 | 1.000147 | 1.004461 | 0.036297 |
| CDKN3 | 1.017528 | 1.008746 | 1.026387 | 8.54E-05 |
| GTSE1 | 1.074797 | 1.048824 | 1.101414 | 7.50E-09 |
| GAL | 1.103696 | 1.028241 | 1.184688 | 0.006319 |
| UNC93A | 1.044995 | 1.011905 | 1.079168 | 0.007344 |
| POSTN | 1.001756 | 1.000577 | 1.002937 | 0.003494 |
| TIMP1 | 1.000233 | 1.000146 | 1.000321 | 1.85E-07 |
| LRG1 | 1.008625 | 1.001193 | 1.016112 | 0.022847 |
| ALDH6A1 | 0.975205 | 0.963947 | 0.986595 | 2.25E-05 |
| CNPY1 | 1.54512 | 1.077485 | 2.215713 | 0.017994 |
| CILP | 1.008463 | 1.00186 | 1.01511 | 0.011926 |
| CDCA7 | 1.06366 | 1.035432 | 1.092658 | 6.88E-06 |
| SLN | 1.009843 | 1.001877 | 1.017872 | 0.015343 |
| RASL11B | 1.007776 | 1.000957 | 1.014642 | 0.02535 |
| APOC2 | 1.281749 | 1.112299 | 1.477014 | 0.000601 |
| PTPN3 | 0.973601 | 0.953925 | 0.993682 | 0.010217 |
| CXCL13 | 1.012036 | 1.00672 | 1.01738 | 8.48E-06 |
| ITIH4 | 1.274029 | 1.113199 | 1.458096 | 0.000436 |
| DGKI | 0.74051 | 0.614073 | 0.892979 | 0.001661 |
| MARCO | 1.009344 | 1.002552 | 1.016181 | 0.006935 |
| PRR11 | 1.052479 | 1.02902 | 1.076472 | 8.69E-06 |
| HRH2 | 0.94325 | 0.916381 | 0.970906 | 7.42E-05 |
| ANGPTL8 | 1.005105 | 1.002506 | 1.007711 | 0.000116 |
| EPYC | 1.009828 | 1.002271 | 1.017441 | 0.010714 |
| TDO2 | 1.046544 | 1.008772 | 1.08573 | 0.015282 |
| RDH12 | 0.861505 | 0.762078 | 0.973905 | 0.017193 |
| CALB2 | 1.078016 | 1.046663 | 1.110307 | 6.08E-07 |
| CLDN10 | 0.974838 | 0.962336 | 0.987502 | 0.000109 |
| WFDC10B | 1.181962 | 1.088519 | 1.283427 | 6.94E-05 |
| SLC5A1 | 0.990307 | 0.984638 | 0.996009 | 0.000883 |
| PLG | 0.972756 | 0.955179 | 0.990657 | 0.002988 |
| TRIM46 | 1.06452 | 1.019147 | 1.111913 | 0.004902 |
| TROAP | 1.062247 | 1.039908 | 1.085066 | 2.57E-08 |
| APOL1 | 1.000569 | 1.000233 | 1.000904 | 0.000888 |
| CCSER1 | 0.364195 | 0.223937 | 0.592301 | 4.69E-05 |
| CLEC4M | 3.969417 | 1.042098 | 15.11976 | 0.043342 |
| COL4A4 | 0.943537 | 0.906521 | 0.982064 | 0.004423 |
| DRGX | 5.767776 | 2.97029 | 11.2 | 2.28E-07 |
| ALX1 | 1.139393 | 1.048889 | 1.237706 | 0.001999 |
| MT1F | 1.005547 | 1.001323 | 1.009788 | 0.010014 |
| SLCO5A1 | 1.954529 | 1.379338 | 2.769577 | 0.000164 |
| KCNK17 | 1.012492 | 1.000392 | 1.024739 | 0.042993 |
| MTURN | 0.962533 | 0.939799 | 0.985817 | 0.00174 |
| SERPINF1 | 1.002075 | 1.001233 | 1.002917 | 1.34E-06 |
| RUNX1 | 1.015862 | 1.005928 | 1.025894 | 0.001696 |
| FAM110C | 0.989823 | 0.983487 | 0.996201 | 0.001798 |
| COL6A3 | 1.002092 | 1.000949 | 1.003237 | 0.000334 |
| PRG4 | 1.008613 | 1.001319 | 1.01596 | 0.020568 |
| SLC17A3 | 0.998844 | 0.997852 | 0.999838 | 0.022651 |
| GXYLT2 | 1.022449 | 1.010556 | 1.034483 | 0.0002 |
| FNDC1 | 1.027165 | 1.001881 | 1.053087 | 0.03505 |
| COBL | 0.891709 | 0.841928 | 0.944433 | 9.21E-05 |
| SLC22A6 | 0.99446 | 0.99004 | 0.998899 | 0.014501 |
| MCOLN3 | 0.884552 | 0.784513 | 0.997347 | 0.045141 |
| SLC6A13 | 0.996804 | 0.994708 | 0.998905 | 0.002888 |
| SLC6A7 | 4.362331 | 1.157649 | 16.43843 | 0.029537 |
| ITIH3 | 1.021686 | 1.001187 | 1.042605 | 0.038017 |
| TUBAL3 | 1.087349 | 1.002288 | 1.179629 | 0.043909 |
| CIDEC | 1.012863 | 1.004509 | 1.021286 | 0.002488 |
| C1R | 1.001743 | 1.000979 | 1.002507 | 7.64E-06 |
| ADH6 | 0.940142 | 0.897464 | 0.984849 | 0.009212 |
| CYS1 | 0.993136 | 0.989857 | 0.996425 | 4.46E-05 |
| NTN4 | 0.99333 | 0.989657 | 0.997017 | 0.0004 |
| NRXN2 | 0.96562 | 0.941291 | 0.990578 | 0.007208 |
| CDC25C | 1.169882 | 1.091459 | 1.253941 | 9.34E-06 |
| GREM1 | 1.041041 | 1.003277 | 1.080226 | 0.032884 |
| AIM2 | 1.053213 | 1.036517 | 1.070178 | 2.03E-10 |
| MYBL2 | 1.018951 | 1.011031 | 1.026934 | 2.42E-06 |
| SUCNR1 | 0.976259 | 0.959428 | 0.993386 | 0.006772 |
| IGFL2 | 1.072381 | 1.022131 | 1.125101 | 0.004319 |
| QRFPR | 0.977812 | 0.964321 | 0.991491 | 0.001548 |
| SLAMF9 | 1.28916 | 1.165323 | 1.426156 | 8.26E-07 |
| GATM | 0.997202 | 0.995252 | 0.999157 | 0.005041 |
| GLYATL1 | 0.987196 | 0.980164 | 0.994279 | 0.000411 |
| IGF2BP3 | 1.091292 | 1.052858 | 1.13113 | 1.79E-06 |
| HIBCH | 0.936428 | 0.910379 | 0.963223 | 5.04E-06 |
| PLPPR4 | 1.092762 | 1.048607 | 1.138776 | 2.49E-05 |
| ENPP2 | 0.99858 | 0.997502 | 0.999658 | 0.009861 |
| PRRX2 | 1.023855 | 1.00517 | 1.042887 | 0.012117 |
| GNB3 | 1.074686 | 1.018571 | 1.133893 | 0.008477 |
| SLC22A13 | 0.911712 | 0.841038 | 0.988326 | 0.024755 |
| SPC24 | 1.042554 | 1.017954 | 1.067749 | 0.000625 |
| KDR | 0.993822 | 0.991361 | 0.996289 | 9.65E-07 |
| HAO2 | 0.987601 | 0.981381 | 0.993861 | 0.000109 |
| ZNRF3 | 0.170003 | 0.033237 | 0.869549 | 0.033352 |
| SORCS1 | 0.631235 | 0.431577 | 0.923262 | 0.017715 |
| COL3A1 | 1.00042 | 1.000088 | 1.000751 | 0.013038 |
| PRUNE2 | 0.987782 | 0.982012 | 0.993586 | 3.91E-05 |
| CDC20 | 1.017795 | 1.011898 | 1.023726 | 2.69E-09 |
| DMRT3 | 1.20638 | 1.060572 | 1.372234 | 0.004307 |
| ATP11A | 0.989395 | 0.984732 | 0.99408 | 9.71E-06 |
| SPACA3 | 1.088022 | 1.007451 | 1.175037 | 0.031629 |
| DDC | 0.993125 | 0.98922 | 0.997046 | 0.0006 |
| GGACT | 0.938455 | 0.899196 | 0.979427 | 0.003575 |
| PITX3 | 1.786864 | 1.005362 | 3.175854 | 0.047907 |
| AQP9 | 1.005493 | 1.000675 | 1.010333 | 0.025382 |
| FAP | 1.07955 | 1.040506 | 1.120059 | 4.65E-05 |
| HOXA13 | 1.034356 | 1.01529 | 1.05378 | 0.000373 |
| DLX4 | 1.314465 | 1.171883 | 1.474394 | 3.05E-06 |
| ACAT1 | 0.989847 | 0.985082 | 0.994636 | 3.40E-05 |
| IRF6 | 0.963386 | 0.944854 | 0.982283 | 0.000167 |
| EBF3 | 0.890925 | 0.826656 | 0.96019 | 0.0025 |
| ARC | 0.821262 | 0.727382 | 0.927259 | 0.001476 |
| KLF17 | 3.182668 | 1.783251 | 5.680284 | 8.96E-05 |
| MNX1 | 1.218734 | 1.048344 | 1.416818 | 0.010042 |
| DACH2 | 0.110546 | 0.014498 | 0.842928 | 0.033601 |
| PCBP3 | 1.052944 | 1.010343 | 1.097342 | 0.014355 |
| CCND1 | 0.99865 | 0.998027 | 0.999274 | 2.25E-05 |
| ENTHD1 | 2.111869 | 1.237172 | 3.60499 | 0.006143 |
| KIF20A | 1.028798 | 1.018503 | 1.039196 | 3.15E-08 |
| DPEP1 | 0.983543 | 0.969254 | 0.998044 | 0.026271 |
| TREM1 | 1.030575 | 1.009078 | 1.052529 | 0.005106 |
| ACHE | 1.017443 | 1.003787 | 1.031284 | 0.012133 |
| SP7 | 3.740973 | 1.372917 | 10.19354 | 0.00989 |
| ROS1 | 1.174714 | 1.100249 | 1.254218 | 1.44E-06 |
| RGS20 | 1.169253 | 1.103208 | 1.239253 | 1.36E-07 |
| MXRA8 | 1.002692 | 1.0011 | 1.004286 | 0.000913 |
| MIOX | 0.998246 | 0.996617 | 0.999877 | 0.035109 |
| ATP8B3 | 1.021764 | 1.002778 | 1.041109 | 0.02446 |
| DLGAP5 | 1.041075 | 1.02457 | 1.057846 | 7.94E-07 |
| FOXM1 | 1.01143 | 1.006741 | 1.016142 | 1.64E-06 |
| WDR72 | 0.958234 | 0.943402 | 0.973299 | 8.30E-08 |
| C8G | 1.059926 | 1.029663 | 1.091079 | 8.23E-05 |
| SLITRK5 | 0.87008 | 0.763811 | 0.991135 | 0.036265 |
| TWIST1 | 1.023363 | 1.009433 | 1.037485 | 0.000958 |
| CCNB2 | 1.033036 | 1.021665 | 1.044535 | 8.66E-09 |
| INHBE | 1.02071 | 1.012063 | 1.02943 | 2.33E-06 |
| TMEM174 | 0.988988 | 0.982082 | 0.995942 | 0.001952 |
| RAB3B | 1.203193 | 1.067 | 1.35677 | 0.002544 |
| PSAT1 | 1.006922 | 1.004059 | 1.009794 | 2.06E-06 |
| TRIML2 | 1.109222 | 1.033157 | 1.190887 | 0.004237 |
| NEUROG3 | 2.799471 | 1.817141 | 4.312841 | 3.03E-06 |
| GDF6 | 0.972744 | 0.956968 | 0.988779 | 0.000924 |
| PTTG1 | 1.018286 | 1.010613 | 1.026016 | 2.65E-06 |
| NLRP6 | 0.951147 | 0.909356 | 0.994858 | 0.0289 |
| HAO1 | 1.203093 | 1.022676 | 1.415339 | 0.025717 |
| TICRR | 1.273392 | 1.183727 | 1.369849 | 8.73E-11 |
| GYG2 | 1.04813 | 1.024117 | 1.072706 | 7.03E-05 |
| MDK | 1.002036 | 1.001108 | 1.002965 | 1.68E-05 |
| SPHK1 | 1.035136 | 1.021913 | 1.04853 | 1.40E-07 |
| C2 | 1.005096 | 1.000273 | 1.009942 | 0.038344 |
| PPARGC1A | 0.968981 | 0.944419 | 0.994181 | 0.01615 |
| C1orf210 | 0.968351 | 0.955162 | 0.981721 | 4.29E-06 |
| TRIM15 | 0.973353 | 0.953681 | 0.993431 | 0.009524 |
| BUB1 | 1.078677 | 1.050582 | 1.107524 | 1.86E-08 |
| HTRA4 | 1.055954 | 1.00146 | 1.113413 | 0.044018 |
| SKA1 | 1.087809 | 1.039587 | 1.138269 | 0.000275 |
| CDKL1 | 0.81946 | 0.730657 | 0.919057 | 0.000668 |
| SSC4D | 1.081216 | 1.007545 | 1.160274 | 0.030103 |
| PCK1 | 0.997123 | 0.994677 | 0.999575 | 0.021506 |
| ADAMTS12 | 1.075463 | 1.027708 | 1.125438 | 0.001693 |
| CENPA | 1.084853 | 1.054699 | 1.115868 | 1.49E-08 |
| ITPKA | 1.023688 | 1.014743 | 1.032712 | 1.71E-07 |
| PKHD1 | 0.95114 | 0.928605 | 0.974221 | 4.23E-05 |
| CD36 | 0.9917 | 0.986409 | 0.99702 | 0.002264 |
| SMIM24 | 0.996909 | 0.995347 | 0.998475 | 0.00011 |
| CTHRC1 | 1.001714 | 1.000942 | 1.002486 | 1.34E-05 |
| SLITRK3 | 7.817564 | 1.111744 | 54.97156 | 0.03879 |
| NR3C2 | 0.91882 | 0.879568 | 0.959824 | 0.000144 |
| AGMAT | 0.990118 | 0.983992 | 0.996282 | 0.001711 |
| DQX1 | 1.100538 | 1.005627 | 1.204407 | 0.03735 |
| SLC17A9 | 1.032004 | 1.010973 | 1.053472 | 0.00271 |
| CGN | 0.936175 | 0.903051 | 0.970513 | 0.000333 |
| WNT4 | 1.08413 | 1.027738 | 1.143616 | 0.003038 |
| USP2 | 0.976205 | 0.962373 | 0.990236 | 0.000941 |
| STOML3 | 0.132096 | 0.032318 | 0.539929 | 0.004833 |
| RSPO4 | 1.086943 | 1.025312 | 1.152279 | 0.005122 |
| CYP4F3 | 1.023727 | 1.009805 | 1.037842 | 0.000789 |
| ATP2B3 | 1.241575 | 1.049775 | 1.468419 | 0.011494 |
| IQGAP3 | 1.087968 | 1.056218 | 1.120671 | 2.41E-08 |
| C1QL1 | 1.001983 | 1.000648 | 1.003319 | 0.003577 |
| PAQR5 | 0.956266 | 0.93996 | 0.972855 | 3.47E-07 |
| PPEF1 | 1.058042 | 1.018805 | 1.09879 | 0.003431 |
| NKX2-2 | 1.211537 | 1.101524 | 1.332537 | 7.79E-05 |
| SAA4 | 1.018487 | 1.005859 | 1.031273 | 0.004007 |
| CEP55 | 1.040283 | 1.023602 | 1.057236 | 1.68E-06 |
| C6orf141 | 1.065371 | 1.025807 | 1.106462 | 0.00104 |
| DLG2 | 0.590719 | 0.349538 | 0.998314 | 0.049268 |
| COL5A1 | 1.002924 | 1.001874 | 1.003975 | 4.62E-08 |
| NCAM2 | 1.543816 | 1.203012 | 1.981165 | 0.000644 |
| CA2 | 0.995268 | 0.992736 | 0.997807 | 0.000263 |
| MMP13 | 1.007011 | 1.002204 | 1.011841 | 0.004215 |
| ZNF189 | 0.983257 | 0.975911 | 0.990659 | 1.02E-05 |
| ANK3 | 0.905819 | 0.872512 | 0.940398 | 2.28E-07 |
| ITIH1 | 1.049632 | 1.014829 | 1.085629 | 0.004869 |
| TLL1 | 0.929796 | 0.894892 | 0.966061 | 0.000192 |
| PTGES | 1.007817 | 1.001711 | 1.01396 | 0.01203 |
| DEPDC1 | 1.07431 | 1.034674 | 1.115464 | 0.000186 |
| ADAM8 | 1.06171 | 1.044453 | 1.079252 | 7.95E-13 |
| SPON2 | 1.004103 | 1.00163 | 1.006583 | 0.001136 |
| MOXD1 | 1.010336 | 1.000737 | 1.020028 | 0.034759 |
| FAM78B | 1.043856 | 1.004925 | 1.084296 | 0.026878 |
