# Supplementary material for: Identification of a basement membrane-based risk scoring system for prognosis prediction and individualized therapy in clear cell renal cell carcinoma
Source: Front Genet. 2023 Feb 3;14:1038924. doi: 10.3389/fgene.2023.1038924 (PMC9935575; doi:10.3389/fgene.2023.1038924)
Supplement: Supplementary file 10 [file Table4.DOCX]

| Table S4. Differential expression analysis of the genes used for BMRS. | | | | | |
| --- | --- | --- | --- | --- | --- |
| gene | conMean | treatMean | logFC | pValue | fdr |
| AJAP1 | 4.337272 | 1.724063 | -1.33098 | 0.005765 | 0.017281 |
| HJURP | 0.143709 | 7.435217 | 5.693151 | 1.49E-05 | 0.000188 |
| P4HA3 | 0.363163 | 2.938557 | 3.016419 | 2.97E-05 | 0.000298 |
| ANK3 | 9.865662 | 3.708661 | -1.41152 | 0.001857 | 0.00703 |
| ADAM8 | 2.699942 | 11.86875 | 2.136168 | 2.97E-05 | 0.000298 |
| CCDC85A | 0.743511 | 1.438813 | 0.952452 | 1 | 1 |
| C8G | 2.470948 | 3.482863 | 0.495209 | 0.378124 | 0.454338 |
